# A Systems Biology Approach Investigating the Effect of Probiotics on the Vaginal Microbiome and Host Responses in a Double Blind, Placebo-Controlled Clinical Trial of Post-Menopausal Women

**DOI:** 10.1371/journal.pone.0104511

**Published:** 2014-08-15

**Authors:** Jordan E. Bisanz, Shannon Seney, Amy McMillan, Rebecca Vongsa, David Koenig, LungFai Wong, Barbara Dvoracek, Gregory B. Gloor, Mark Sumarah, Brenda Ford, Dorli Herman, Jeremy P. Burton, Gregor Reid

**Affiliations:** 1 Canadian Centre for Human Microbiome and Probiotic Research, Lawson Health Research Institute, London, Canada; 2 Microbiology and Immunology, The University of Western Ontario, London, Canada; 3 Kimberly Clark Corporation, Corporate Research and Engineering-Microbial Control, Neenah, Wisconsin, United States of America; 4 Biochemistry, The University of Western Ontario, London, Canada; 5 Agriculture and Agri-Food Canada, London, Canada; 6 Springbank Medical Clinic, London, Canada; 7 Surgery, The University of Western Ontario, London, Canada; 8 Division of Urology, The University of Western Ontario, London, Canada; GI Lab, United States of America

## Abstract

A lactobacilli dominated microbiota in most pre and post-menopausal women is an indicator of vaginal health. The objective of this double blinded, placebo-controlled crossover study was to evaluate in 14 post-menopausal women with an intermediate Nugent score, the effect of 3 days of vaginal administration of probiotic *L. rhamnosus* GR-1 and *L. reuteri* RC-14 (2.5×10^9^ CFU each) on the microbiota and host response. The probiotic treatment did not result in an improved Nugent score when compared to when placebo. Analysis using 16S rRNA sequencing and metabolomics profiling revealed that the relative abundance of *Lactobacillus* was increased following probiotic administration as compared to placebo, which was weakly associated with an increase in lactate levels. A decrease in *Atopobium* was also observed. Analysis of host responses by microarray showed the probiotics had an immune-modulatory response including effects on pattern recognition receptors such as TLR2 while also affecting epithelial barrier function. This is the first study to use an interactomic approach for the study of vaginal probiotic administration in post-menopausal women. It shows that in some cases multifaceted approaches are required to detect the subtle molecular changes induced by the host to instillation of probiotic strains.

**Trial Registration:**

ClinicalTrials.gov NCT02139839

## Introduction

The vaginal microbiota is a dynamic ecosystem that is usually mono dominated by the *Lactobacillus* genus in times of health but it can transform quickly to a dysbiotic state where a range of microorganisms rise in prominence and cause the polymicrobial bacterial vaginosis (BV) [1]. The pre-menopausal vaginal microbiota and its constituents have been extensively studied using genome sequencing, 16S rRNA community profiling and RNA-seq based meta-transcriptomics [2]–[5]. However, until recently, few studies have used similar in-depth methods to decipher the vaginal microbiome of post-menopausal women, despite its impact on quality of life [6], [7]. In previous studies, we have shown using high-throughput sequencing methods that contrary to conventional knowledge [8], the vaginal microbiome of post-menopausal women is not significantly different from that of pre-menopausal women [9]. Our study showed that the post menopausal vagina appeared to have greater stability than the rapidly fluctuating pre-menopausal microbiome [9], [10] presumably due to a lack of hormone cycling. We found that similar to premenopausal women, the healthy post-menopausal vaginal microbiome is dominated by a combination of *Lactobacillus iners* and *L. crispatus* while BV-associated organisms such as *Gardnerella vaginalis* and *Atopobium vaginae* are increased in states of dysbiosis [9], [11], [12]. Despite some deficiencies, Nugent scoring is still the standard technique in most research and clinical settings to rapidly diagnose bacterial vaginosis [13]. The score is calculated by microscopic examination of a Gram-stained vaginal smear and numeration of cell morphotypes to assign a score from 0 to 10 where 0–3 is considered normal, 4–6 is intermediate and 7–10 is BV [13]. The intermediate scores are particularly interesting as they indicate a risk of transition to BV, or they could be a state that reverts to a healthy lactobacilli dominated microbiota.

Given vaginal contact with sanitary products by menstruating women and the large proportion of postmenopausal women whom suffer urinary incontinence requiring products such as incontinence pads, also in proximity to the vagina, these products may offer a vehicle to deliver a prophylactic probiotic to women that routinely suffer BV or urinary tract infections. We were interested to determine whether the microbiota and Nugent scores were altered by the administration of probiotics with the potential view of perhaps instilling these organisms in such products. *Lactobacillus rhamnosus* GR-1 and *Lactobacillus reuteri* RC-14 used in this prospective study are well-characterized probiotic strains used in combination to prevent and treat BV [14]–[17]. In addition to clinical and Nugent scoring, 16S rRNA sequencing was used to examine changes in the microbiota, GC-MS profiled metabolome changes, the host transcriptional responses were tested using an Affymetrix microarray and inflammatory mediators by multiplex cytokine analysis. This approach was designed to provide a holistic understanding of the probiotic-microbiota-host interactome [18].

## Results

### Participant recruitment and demographics

**Figure 1:**
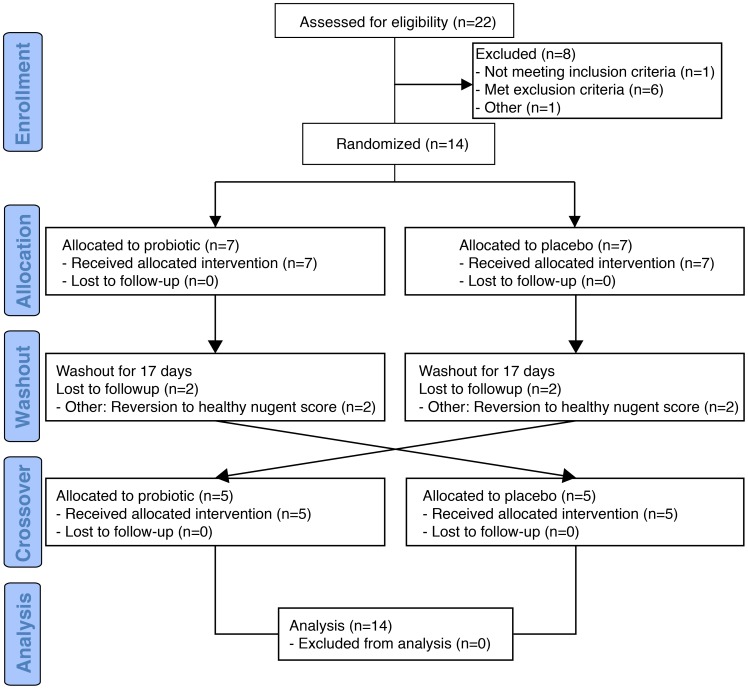
CONSORT flow diagram.

A total of 22 subjects were screened and 14 participants were enrolled into a 129-day prospective double-blind, cross-over placebo controlled study (design in Figure S1). A CONSORT flow diagram is displayed in Figure 1.

### Clinical Outcomes

Nugent score and vaginal pH measurements (data not shown) were taken before and after treatment with the probiotic and placebo. Table 1 shows there were no statistically significant improvement in Nugent scores between the treatment groups. Furthermore no statistical differences were observed in vaginal pH measurement.

**Table 1:** Summary of Nugent Score Change - Proportion of Improvement from Baseline.

			Placebo		
			Improved^1^	Not Improved	*p*-value^2^
One-Day after last Test Article Use	Probiotics:	Improved	0	2(20%)	0.1573
		Not Improved	0	8(80%)	
Eight-Days after last Test Article Use	Probiotics:	Improved	0	2(20%)	0.5637
		Not Improved	1(10%)	7(70%)	

1 Improvement in Nugent Score: indicated by shift of intermediate score (4–6) at baseline to normal score (0–3) at follow-up visits. Baseline Nugent score for study Phase-I was collected at visit 3, and baseline Nugent score for study Phase-II was collected at visit 6.

2 *p*-value is based on McNemars test to determine results of cross-over treatment regimen.

### Microbiome profiling

Sequences were successfully obtained for 104 of 106 samples with the mean number of reads being 33,396 (range 1,475 to 223,629) across 3 separate 316 Ion Torrent Sequencing Chips. A total of 221 Operational Taxonomic Units (OTUs) were defined with clustering at 97% identity (Table S1) at a minimum abundance of 1% in any one sample. A no template control was also sequenced and high-abundance contaminating sequences were filtered out *in silico* before OTU assignment.

**Figure 2:**
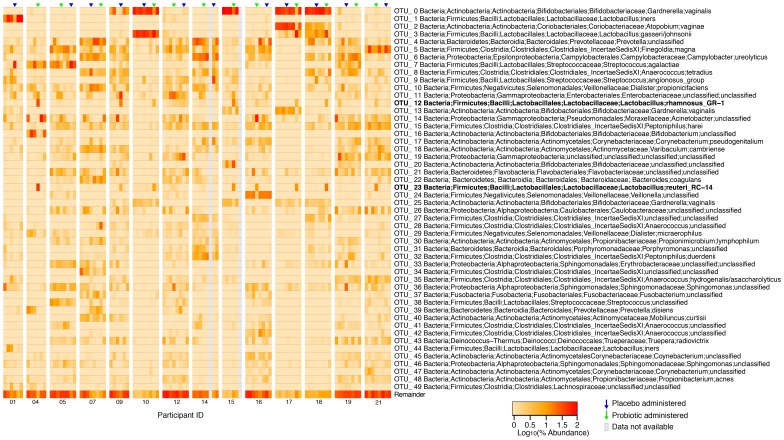
Microbiota heat map. Each column represents the microbiota of a single sample with the 50 most abundant OTUs displayed with their taxonomies and the remainder pooled. Samples are clustered by the participant of origin and organized from first to last visit from left to right. The time points immediately following administration of the placebo or probiotic are indicated with a blue or green arrow (respectively). OTUs representing the putative OTUs for *L. rhamnosus* GR-1 and *L. reuteri* RC-14 have been bolded.

Figure 2 shows a heat map of the 50 most abundant OTUs across all samples and time points. We were able to identify 2 OTUs which were assigned presumptive strain designations to *L. rhamnosus* GR-1 and *L. reuteri* RC-14 (OTU_12 and OTU_23 respectively) based upon 100% ID matches to the 16S rRNA gene of private genome assemblies of these strains. A separate heat map was created showing only the abundance of these two OTUs compared to all other OTUs detected (Figure S2). Furthermore with one notable exception (participant 19), these strains only appeared in combination following the probiotic treatment period. In the case of participant 19 however, the proportion of reads is still quite low (2.1% and 0.1%) for both OTUs. The *L. rhamnosus* GR-1 is far more abundant than the *L. reuteri* which may suggest it was a *L. casei* group member other than the GR-1 used in the study as this group has high sequence homology, especially in the V6 16S rRNA region [19]. In one individual (participant 09) both probiotic strains were detected even though only placebo should have been received. The probiotic strains could be unambiguously detected after probiotic treatment in 7 of 12 cases (04, 10, 15, 16, 17, 18, 21), which we termed responders. While in the non-responders (12, 14, 19, 05 and 07) the probiotic strains were not detected after the probiotic treatment. The abundance of *L. rhamnosus* GR-1 did appear to increase over the study period in participant 12, which may suggest temporary persistence or colonization. In all other cases, the probiotics were only detected in high abundance immediately after the treatment suggesting that the washout period chosen for the study was appropriate.

**Figure 3:**
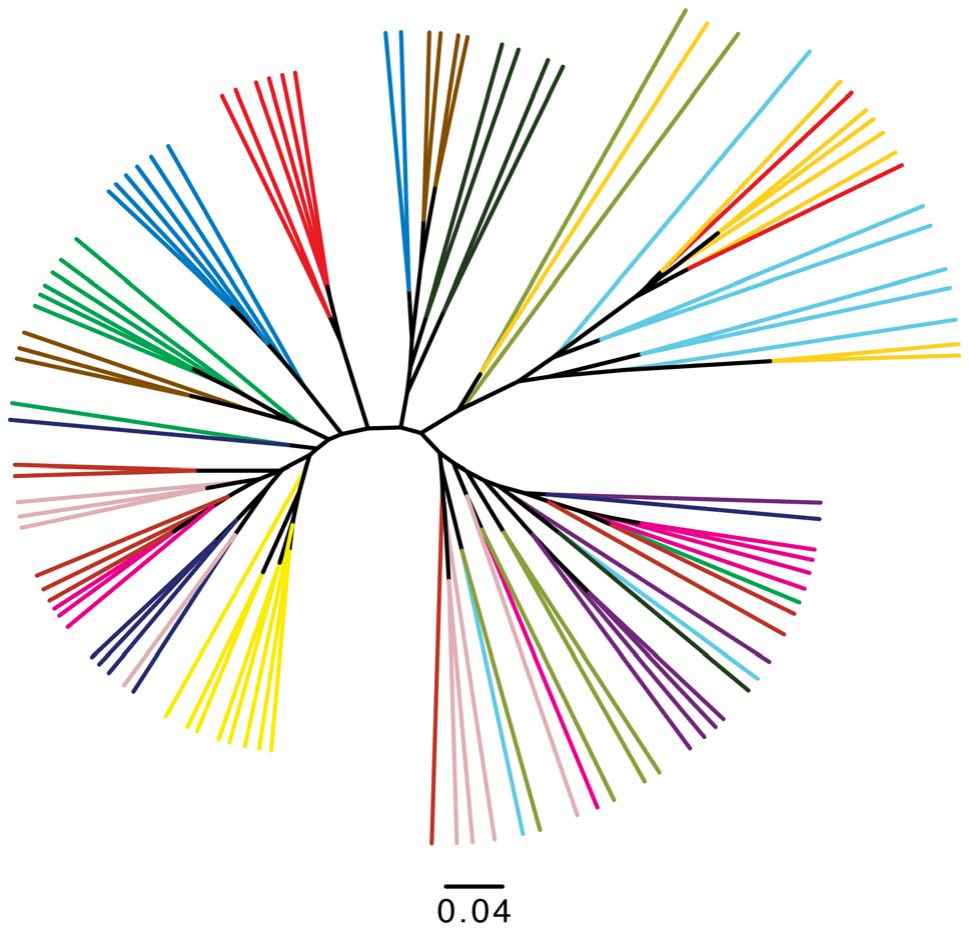
UPGMA clustering of all participants microbiota based upon weighted UniFrac distances. In general, participants cluster most closely with themselves. The sample tips are colored by the participant of origin.

Samples were clustered based upon unweighted pair group method with arithmetic mean (UPGMA) using weighted UniFrac distance metrics [20] showing that participants were generally most similar to themselves over the study period and as we had previously shown, the post menopausal vaginal microbiome is more stable than that of pre menopausal women (Figure 3).

All samples were plotted on a PCoA using weighted Unifrac distances and overlaid with the contributions of the 20 most abundant genera (Figure S3). This shows 3 weak groupings, one dominated by *Lactobacillus*, another defined by *Atopobium* and *Gardnerella*, and a third defined by a large number of diverse genera.

**Figure 4:**
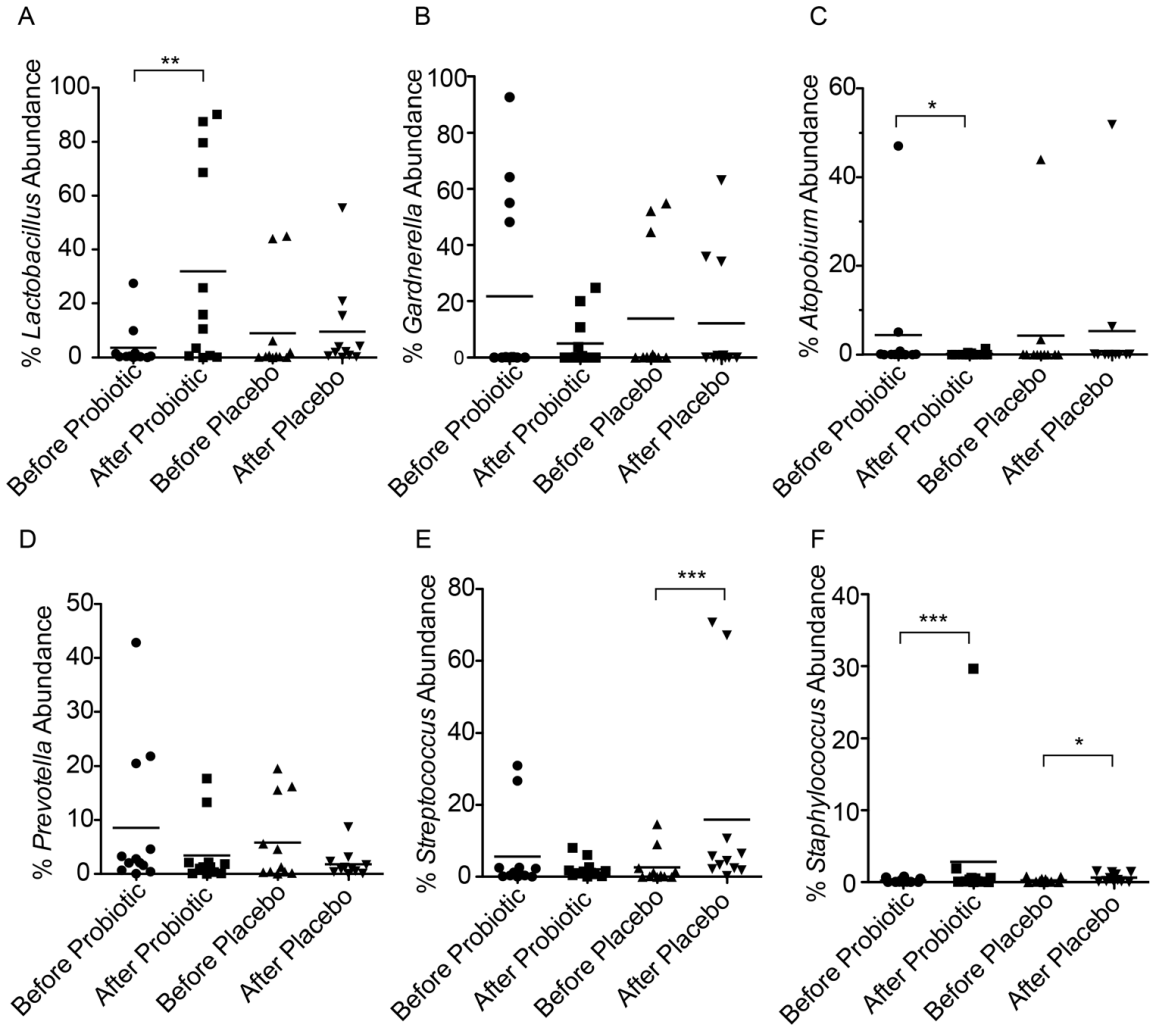
Selected genus relative abundances following probiotic and placebo interventions. (A) *Lactobacillus*, (B) *Gardnerella*, (C) *Atopobium*, (D) *Prevotella*, (E) *Streptococcus*, (F) *Staphylococcus*. Following probiotic administration for 3 days, the proportion of *Lactobacillus* is significantly increased while that of *Atopobium* is decreased and *Staphylococcus* is increased. Placebo interventions increased *Streptococcus* and *Staphylococcus* abundance. *FDR<0.1, **FDR<0.05, ***FDR<0.01.

**Figure 5:**
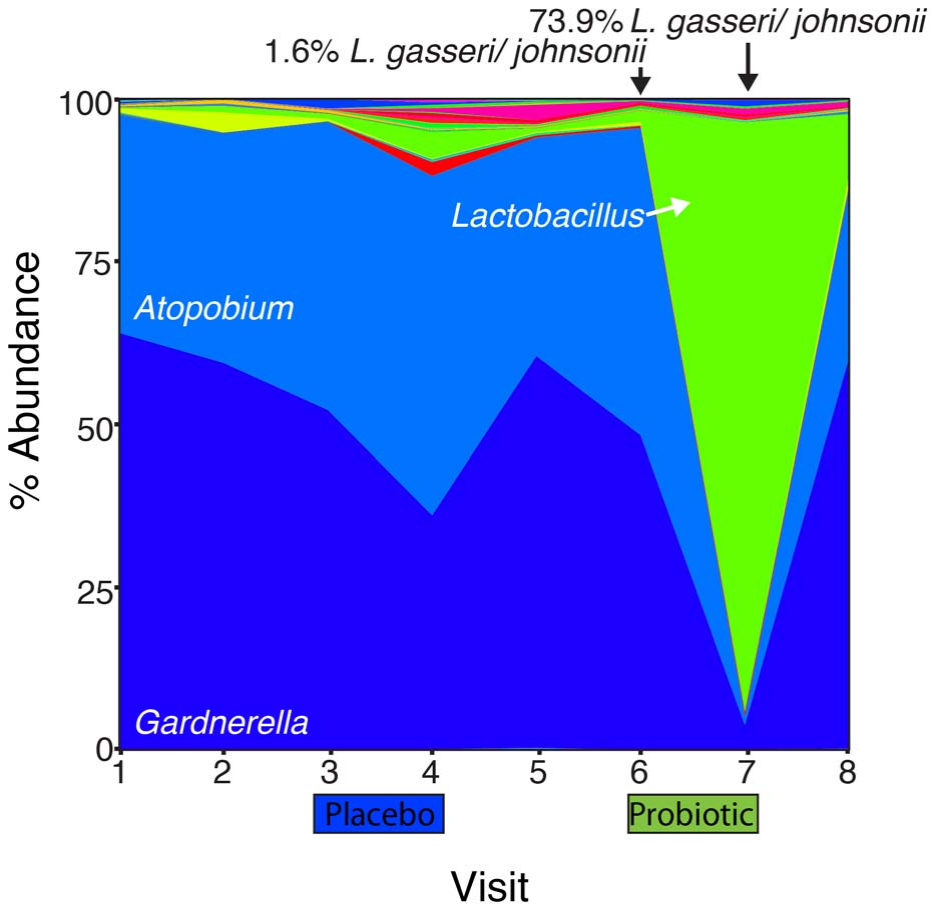
Time series of participant 17. During the administration of probiotic between visits 6 and 7, the abundance of lactobacilli significantly increases, decreasing the proportional abundance of both *Atopobium* and *Gardnerella*. This increase is not due solely to the probiotic strains as a significant increase in indigenous *L. gasseri*/*johnsonii* takes place.

Data were summarized to genus level and the relative abundance of *Lactobacillus* was considered. Increase in total *Lactobacillus* is significant (Figure 4; FDR<0.05) following probiotic administration though there is no significant difference in Shannon's diversity index (p = 0.25, Wilcoxon signed-rank test). After probiotic administration, the proportion of total lactobacilli increased in all participants except participant 19 who appears to have had an indigenous *L. casei*/*rhamnosus* present. Even those without significant detection of the probiotic lactobacilli have an increase in indigenous lactobacilli (fold increases for the ambiguous participants are 1.27, 1.21, 2.35, 14.43 and 52.43). Additionally, in some cases, such as participant 17 (Figure 5), it is not only the probiotics that increased in proportional abundance, but also indigenous lactobacillus such as *L. gasseri/johnsonii*, (p = 0.011 across all samples) which is not significant in the placebo group if the case of participant 09 is excluded (where probiotic appears to have been received rather than placebo). In the 7 individuals where the probiotics could be easily detected, the average fold increase was 120 fold (range 3.2 to 389). Significant decreases in the proportion of *Atopobium* were also observed after probiotic treatment with a trend of reduction of *Gardnerella* and *Prevotella*. Interestingly the placebo appears to have increased the proportion of *Streptococcus* (FDR<0.01) while both applications appear to have increased levels of *Staphylococcus*.

### Metabolome

Using GC-MS, 68 metabolites were detected and a heat map is displayed in Figure S4. The Pearson's correlation coefficient between lactate and lactate producing bacteria was 0.47 (p<0.001), indicating a weak positive correlation (Figure S5). Among those who responded to probiotic intervention and had sufficient material for metabolomics analysis (n = 4), there was an increase in lactate levels (p = 0.13), whereas there tended to be a decrease with placebo (p = 0.23). There was no difference in the overall metabolome of all subjects after probiotic or placebo intervention (Figures S6A and S6B respectfully), nor was there a significant difference in any one metabolite. It is worth noting that low yields of metabolites were obtained from some of the collected swabs so lower abundance analytes may not be detectable.

### Cytokine/Chemokine analysis

Interleukin 5 (IL-5) was found to be significantly up-regulated following probiotic treatment but not following placebo treatment. In other cytokines such as IL-1B, IL-6, TNFa and GM-CSF there were weak positive trends in increase of cytokine levels, however this was also observed in the placebo in some cases (Figure S7).

### Microarray

Due to limited yield from RNA samples, only 2 complete sample sets (before and after intervention from the same individual, 4 arrays) were obtained from the probiotic intervention and 3 sets from the placebo intervention (6 arrays). Each sample was analyzed on its own Affymetrix Human Gene 2.0 ST array. Using a paired t-test design to account for paired-samples before and after probiotic administration, 90 probe sets were detected as being differentially expressed (≥2 fold change, p<0.05) of which 57 were coding genes or long non-coding RNA (a new feature of the Affymetrix 2.0 ST array) (Table S2A). Two of these were differentially regulated in both conditions (gene list differentially regulated by placebo is displayed in Table S2B), ANKRD20A5P psuedogene (ID cluster 16851230) and a long non-coding RNA (ID cluster 16851230) with no known biological significance.

Many genes of interest were differentially expressed by probiotic treatment including Interleukin 18 (IL18; −2.5 fold after probiotic), CR1-complement receptor (CR1; 10.4 fold), Caspase 14 (CASP14; 5.3 fold, previously observed to function in epithelial development and barrier function [21]) and Toll like receptor 2 (TLR2; 3.84 fold) (Table S2A). To look at functional consequences, GO enrichment was applied showing the most highly affected functions to be pattern recognition receptor activity, complement receptor activity, inflammatory response and gram-positive bacterial cell surface binding (Table 2). This was mirrored in the Ingenuity Pathway Analysis (Figure S8). In comparison, the placebo appears to have acted on functions related to epithelial cell differentiation, similar to our previous study [9].

**Table 2:** Gene Ontology Enrichment in genes differentially expressed by probiotic intervention.

Function	Enrichment Score	Enrichment p-value	# genes in list	% genes in group	GO ID
Pattern Recognition Receptor Activity	12.82	2.71E-06	3	18.75	8329
Complement Receptor Activity	10.9319	1.79E-05	2	50	4875
Inflammatory Response	10.4433	2.91E-05	6	1.70455	6954
Gram-positive Bacterial Cell Surface Binding	10.4222	2.98E-05	2	40	51637
LPS Receptor Activity	10.4222	2.98E-05	2	40	1875
Defense Response	9.64889	6.45E-05	9	0.840336	6952
Regulation of GM-CSF Production	8.92362	1.33E-04	2	20	32645
Positive Regulation of Response to External Stimulus	8.83166	1.46E-04	4	2.5974	32103
Response to Wounding	8.61585	1.81E-04	6	1.22449	9611
Positive Regulation of Inflammatory Response	8.25985	2.59E-04	3	4.22535	50729

Genes differentially expressed (≥2-fold change, p<0.05) were subjected to GO enrichment analysis. Functions heavily relate to innate immune system responses including responses to both Gram-positive and Gram-negative bacteria.

## Discussion

In this study, we aimed to evaluate the effect of 3 days of vaginal administration of *L. rhamnosus* GR-1 and *L. reuteri* RC-14 on the nugent score of post-menopausal women as well as effects on the microbiota, metabolome and host. As the subjects under study had no symptoms of disease, not unexpectedly, the probiotic prophylaxis did not cause any clinical changes. There were no adverse effects of the probiotic therapy. The probiotic administration resulted in a temporary increase in the relative proportion of *Lactobacillus* in the vagina, which for women whose intermediate score tends to be a predecessor for BV, may help prevent or delay this transition. The near absence of *L. crispatus* (OTU_198), detected in only 1 of 15 women above a 1% threshold and throughout the study at maximum 1.4%, may indicate that the subjects were at a higher risk of BV, since *L. crispatus* is thought to play a protective role in maintaining the vaginal microbiota [22].

The increase in lactobacilli and potential decreases in BV-associated organisms such as *Atopobium* following probiotics could have been due to displacement of the host's microbes with the total bacterial load remaining the same; or the total bacterial load increased as a result of adding in exogenous lactobacilli. The observation that Shannon's diversity is not affected by probiotic instillation favors the second option. It is interesting to note the increased relative abundance of *Staphylococcus* as a result of placebo treatment, which may be the result of the antimicrobial actions of titanium dioxide used in the preparation. It is important to note that the probiotics may create an environment conducive to indigenous lactobacilli such as *L. gasseri* and *L. johnsonii*, however this effect may be short lived. A longer treatment time and follow-up might have determined if there was an effect on diversity, but we were interested in whether a short term probiotic application that women might find easy to administer monthly, could increase the lactobacilli count.

In some individuals it was extremely clear when the probiotic strains had been applied. The reason for this not being universal is unclear, but could be due to a particularly resilient indigenous microbiota or women who were non-responders for unexplained reasons [23].

We identified a positive correlation between lactate abundance and percentage of lactate producers present r = 0.43 (p = 9.6×10^−6^). Although this correlation seems intuitive, to our knowledge it has never been directly shown. Only the relative abundance of bacterial taxa are measured, and therefore absolute changes affected lactate levels cannot be directly observed. Certain species of *Lactobacillus* have been associated with lower pH and may produce more lactate compared to others [3]. There was a trend towards an increase in lactate after probiotic and decrease after placebo intervention, but as there were only four women whom responded to treatment and from whose samples enough material for metabolite analysis could be extracted, the sample size is likely too small to reach significance. Still, the observation that probiotics could potentially increase lactate levels is promising as lactate has been shown to have many beneficial properties in the vaginal tract such as HIV inactivation [24].

Overall, the administration of the two lactobacilli strains did not induce any more changes than placebo in the metabolome, but lack of material makes this result inconclusive. In our experience and that of others, the metabolomic patterns differ between health and BV, with the latter showing odorous compounds such as cadaverine and putrescine, but these trends were not clearly observed in this study [25].

In addition to looking at the bacterial communities, we sought to examine host responses to probiotic treatment. It has been well established that probiotic strains can affect host transcription in the gut in a strain specific manor [26] but this is the first time similar studies have been carried out in the vagina. Our findings show that *L. rhamnosus* GR-1 and *L. reuteri* RC-14 had an immunomodulatory effect working on important central inflammatory mediators complement receptor 1, toll like receptor 2 and IL-18. Though limited in the number of subjects available for analysis, the strength of this analysis is the use a paired study design. Interleukin 18 is a proinflammatory cytokine inducing cell-mediated immunity via interferon gamma though it also has effects on B cells and IgE production [27]. In the female reproductive tract it may be of relevance in preterm birth [28] indicating microbial invasion of the amnion, but it also has protective roles against genital herpes simplex 2 [29]. The effect of probiotic treatment on IL-18 expression may not be a direct interaction, but rather indicative of anti-viral actions of the probiotics resulting which has previously been hypothesized and shown *in vitro* [24], [30]. Alternatively it may reflect a general up-regulation of the innate immune system by the probiotic strains. Unfortunately, our cytokine/chemokine panel did not cover IL-18 to help support this finding. Alternatively the up-regulation, of complement receptors 1 and 3a, as well as TLR2 both are indicative of increased anti-bacterial innate immune system activity. TLR2 specifically is involved in detection of Gram-positive surface markers (including lactobacilli) [31] while the complement receptors are important for phagocytosis and inflammatory cascades in response to bacterial infections [32]. *L. plantarum* has previously been shown to modulate TLR2 expression which may play a role in modifying infection response [33]. In addition, our cytokine/chemokine multiplex results may indicate the up-regulation of interleukin 5 which is most commonly associated with eosinophil activation [34]. Though commonly associated with parasites, eosinophils have also been associated with *Actinomyces*-like organisms in the urogenital tract and may display some activity against bacterial infection [35]. These seemingly pro-inflammatory responses may be beneficial in aiding the host immune system to help clear BV-associated pathogens to restore a more normal microbiota of symbionts. Microarray analysis failed to collaborate the finding that interleukin 5 was up-regulated, however the two individuals surveyed by microarray did not show up-regulation by cytokine analysis either. If all participants could have been surveyed this result may have been different.

Interestingly, caspase 14 plays a role in development and barrier function of the epidermis [21] and we had previously observed its differential regulation associated with vaginal dryness and dysbiosis in a subset of post-menopausal women. Its up-regulation following probiotic treatment may be evidence of improvement in strength and integrity of the vaginal epithelium, though further research is needed to verify this claim. Indeed probiotics are thought to positively act on barrier function [36].

In conclusion, this study demonstrated the use of a systems-wide interactomics approach to examine how probiotic application might modulate the vagina in post-menopausal women. The short duration therapy increased the lactobacilli, as well as modulating inflammatory markers. As the transition from healthy to intermediate to BV is such a common occurrence, women may feel that a three day intravaginal probiotic may be beneficial. A larger study is needed to support this, but the present study showed no harm and the potential for such a benefit.

## Methods

### Participant Recruiting and study design

The protocol for this trial and supporting CONSORT checklist are available as supporting information; see Checklist S1 and Protocol S1. A total of fourteen post-menopausal women were enrolled in the study from a clinical site in Ontario, Canada. Inclusion criteria were individuals with an intermediate Nugent Score (4–6) who were post-menopausal (40 to 80 years old, not having had a menstrual period for the last 12 months, currently in a mutually monogamous sexual relationship or not sexually active, agreeing to be abstinent 72 hours prior to each study visit and agreeing to refrain from intercourse for 48 hours after treatment, agreeing to abstain from use of other intravaginal products throughout the study and in good general health. Exclusion criteria were use of intravaginal products within three months prior to visit 1, a history of immunosuppressive drug therapy, chemotherapy or radiation therapy, a medical condition which might compromise immune system function (ex. cancer, leucopenia, HIV, organ transplant), antibiotic and/or antifungal medication within the last 4 weeks, oral probiotic use within 3 months prior to visit 1, significant changes in diet during the course of the study (based on self report), induced menopause due to surgical/medical intervention (ex. hysterectomy), currently taking estrogen therapy, a history of drug or alcohol abuse, known allergy to either of the probiotic strains or product excipients and participation in a clinical trial involving an investigational product/device within the past three months or who are scheduled to participate in another clinical study concurrently. No formal sample size calculation was carried out and the number of subjects to be randomized was based on feasibility and practical considerations for an exploratory study. The study was approved by Health Canada (Clinical Trial Application File No. 180061) and is registered with clinicaltrials.gov under accession number (NCT02139839).

The study design is diagramed in Figure S1. Briefly, after obtaining written informed consent, participants were enrolled on day one and subsequently returned for baseline appointments on days 5 and 15 (visits 2 and 3 respectively). On visit 3, the participants were randomized to either receive 3 days of *L. rhamnosus* GR-1/*L. reuteri* RC-14 probiotic (Chr. Hansen, minimum 2.5×10^9^ CFU of each strain) or visually identical placebo (49 mg Gelatin and 1 mg titanium dioxide) self-administered by having the participant insert the capsule as far as comfortable into their vagina and then lying in a horizontal position for 15 minutes. Randomization was carried out using a random number generator. Both study staff and study participants were blinding as to the study treatment being given. The capsules were to be administered twice a day for 3 days with the participant returning on the fourth day for sampling. A feminine hygiene pad was applied for several hours after capsule implantation. There was then a 17 day washout period with sampling again at day 26. At day 36 participants were again sampled, individuals who received probiotic in the first treatment period then received placebo and vice versa. On day 40, participants returned for sampling after the treatment period and were followed up with on day 47 and again on day 129, only if they had positive culture results for the presence of the probiotic. At all visits 5 swabs were collected: (i) Dacron swab for bacterial DNA extraction, (ii) Dacron swab for multiplex cytokine/chemokine analysis, (iii) Dacron swab for metabolomics analysis, (iv) a cytobrush (Cytobrush plus GT, Cooper Surgical Inc. USA) brushed against the vaginal wall and stored in 700 µL RNAlater for total RNA extraction (Life Technologies), and (v) Dacron swab for Nugent Score. McNemar's test was applied to examine changes in Nugent score and other symptoms.

### Microbiome profiling

Vaginal swabs for microbiome analysis were extracted using the QIAamp DNA stool mini kit (Qiagen). Swabs were vortexed in 1 mL buffer ASL before removal of the swab and addition of 0.1 mm zirconia/silica beads (Biospec Products) with 2, 30 second rounds of bead beating at full speed with cooling on ice in between (Mini-BeadBeater; Biospec Products). Sample amplification for sequencing was carried out using the forward primer (CCATCTCATCCCTGCGTGTCTCCGACTCAGxxxxxCWACGCGARGAACCTTACC) and the reverse primer (CCACTACGCCTCCGCTTTCCTCTCTATGGGCAGTCGGTGATACRACACGAGCTGACGAC) where xxxxx was a sample specific nucleotide barcode, the 5′ end is the adapter sequence for the Ion Torrent sequencer and the sequences following the barcode are complementary to the V6 rRNA region. Amplification was carried out in 42 µL with each primer present at a 10 µL (3.2 pMol/µL stock), 20 µL GoTaq hot start colorless master mix (Promega) and 2 µL extracted DNA. The PCR protocol was as follows: initial activation step at 95°C for 2 minutes and 25 cycles of 1 minute 95°C, 1 minute 55°C and 1 minute 72°C. PCR products were quantified with a Qubit 2.0 Flourometer and the high sensitivity dsDNA specific fluorescent probes (Life Technologies). Samples were mixed at equimolar concentrations and purified with the QIAquick PCR Purification kit (QIAGEN).

All subsequent work was carried out at the London Regional Genomics Centre (LRGC, lrgc.ca, London, Ontario, Canada). Briefly, samples were prepared with an Ion OneTouch System (Life Technologies) and sequenced on an Ion Torrent Personal Genome Machine sequencer on a 316 chip (Life Technologies).

Resulting Reads were extracted and de-multiplexed using modifications of in-house perl and unix-shell scripts [9] with OTUs clustered at 97% identity. Table S3 displays the nucleotide barcodes and it's corresponding sample. Reads were deposited to the Short Read Archive (BioProject ID: PRJNA244441). To control for background contaminating sequences, a no-template control was also sequenced, and any individual sequence unit belonging to an OTU present at ≥1% abundance in the NTC was removed before re-clustering with uclust 3.0.612.

Automated taxonomic assignments carried out by examining best hits from comparison the Ribosomal Database Project (rdp.cme.msu.edu) and manually curated as before [9].

Alpha and beta-diversity analysis were made using the OSX distribution of QIIME (MacQIIME 1.70, wernerlab.org/software/macqiime). OTU tables were rarified to 1060 reads for all analysis to control for uneven sampling depth. To avoid inappropriate statistical inferences made from compositional data, log-ratios, a method previously described by Aitchison and adapted to microbiome data was used [37]–[39] with paired t-tests for comparisons of genus and species level data. The False Discovery Rate (FDR) method was used to control for multiple testing with a significance threshold of FDR = 0.1. All statistical analysis, unless otherwise indicated, was carried out using R (r-project.org).

### Microarray analysis

Vaginal cytobrushes were extracted as previously [9]. Briefly a vaginal cytobrush was centrifuged at 5000×*g* for 10 min at 4°C and the supernatant discarded. The pellet was then extracted with TRIzol (Life Technologies) following the manufacturer's protocol before being DNase treated with Turbo DNA-free kit (Life Technologies). Sample quantity and quality were determined using a Nanodrop 1000 spectrophotometer (Thermo Fisher Scientific, Waltham, USA) and Agilent 2100 Bioanalyzer (Agilent Technologies, Santa Clara, USA). Samples were prepared for analysis on the GeneChip Human 2.0 ST array (Affymetrix, Santa Clara, USA) at the LRGC following standard protocols.

Probe level data was imported into Partek Genomics Suite (Partek, St. Louis, USA) using the RMA algorithm. Gene expression data was deposited in the Gene Expression Omnibus Database with the accession number GSE54363.

To study the effects of the probiotic and placebo treatments on host gene expression, and considering the study design, a paired t-test was used to evaluate gene expression changes in each individual immediately before and after probiotic/placebo administration. This yielded two individuals (14 and 16) with arrays immediately preceding and following the probiotic period treatment and three individuals (01, 14, 05) with arrays surrounding the placebo treatment period. For other participants, insufficient yield and/or quality resulted in a lack of paired samples, and they were thus excluded from downstream analysis.

### Sample Preparation GC-MS

Samples were collected from the mid-vaginal wall using the Cytobrush vaginal brush. Vaginal brushes were pre-cut into 1.5 mL tubes and weighed prior to and after sample collection to determine the mass of vaginal fluid collected. Samples were stored at −80°C until analysis. After thawing, brushes were cut and eluted in methanol-water (1∶1) in 1.5 mL microcentrifuge tubes. The weight of each sample was divided by the weight of the lightest sample and this fraction was multiplied by 200 µL to determine the volume of methanol-water to add to each sample. This corresponded to a volume of 200–382 µL, depending on the mass of vaginal fluid collected. A blank swab eluted in 200 µL methanol-water was included as a negative control. All samples were vortexed for 10 s to extract metabolites, centrifuged for 5 min at 10 000×*g*, vortexed again for 10 s after which time the brushes were removed from tubes. Samples were centrifuged a final time to pellet cells and 150 µL of the supernatant was transferred to a GC-MS vial. Next, 2.5 µL of 2 mg/mL ribitol was added to each vial as an internal standard. Samples were then dried to completeness using a SpeedVac. After drying, 100 µL of 2% methoxyamine•HCl in pyridine (MOX) was added to each for derivatization and incubated at 50°C for 90 min. 100 µL N-Methyl-N-(trimethylsilyl) trifluoroacetamide (MSTFA) was then added to each vial and incubated at 50°C for 30 min, then transferred to micro inserts before running on GC-MS (Agilent 7890A GC, 5975 inert MSD with triple axis detector, 30 m DB5-MS column with 10 m duraguard column). Samples were analyzed once for untargeted whole metabolome analysis, and a second time on selective ion monitoring (SIM) mode specific for lactate using the reference ions 117, 147, 191 and 219. Solvent delay was 10 min and sample injection volume was 1 uL. All samples were run in random order and one was run multiple times throughout to ensure machine consistency.

### Lactate relative abundance quantification

Chromatograms were deconvoluted in Chemstation (Agilent) and lactate relative abundance determined by peak area using the auto integration function. Lactate relative abundance was plotted against the percentage of lactate producers (*Lactobacillus*, *Bifidobacteria*, *Atopobium*, *Streptococcus*, *Staphylococcus*, *Weisella*) present in each sample to determine the correlation between lactate levels and lactate producing bacteria. The correlation value was determined in R using a Pearson's correlation. Paired t-tests were used to determine if probiotic or placebo intervention had a significant effect on lactate levels using a significance threshold of p = 0.05.

### Whole metabolome analysis

Chromatogram files were converted to ELU format using the AMDIS Mass Spectrometry software [40]. Chromatograms were then aligned and abundance of metabolites calculated using the Spectconnect software [41], with the support threshold set to low. In order to determine changes in the metabolome due to probiotic or placebo, Principle component analysis (PCA) was conducted in SIMCA (Umetrics) using the relative abundance matrix (RA) output from Spectconnect. Data were mean centered and pareto scaled prior to PCA. A Mann- Whitney U test was then used to determine metabolites that were significantly altered by intervention (p<0.05).

### Cytokine/Chemokine Measurement

Swabs were resuspended in 200 µL extraction buffer (20 mM Tris-HCl ph7.5, 150 mM NaCl, 1 mM PMSF, 0.05% Tween 20 and 1 uL/mL protease inhibitor cocktail (Roche), vortexed, incubated overnight at 4°C, swab removed and then 50 µL more extraction buffer was added before being stored at −80°C. The resulting samples were thawed on ice and loaded onto a Milliplex Human High Sensitivity Cytokine/Chemokine Panel (EMD Millipore, Billerica, Mass, USA) and analyzed for IL-1B, IL-2, IL-4, IL-5, IL-6, IL-7, IL-8, IL-10, IL-12p70, IL-13, IFNg, GM-CSF, and TNFa. Results for IL-2, IL-7, IL-8, IL-10, IL-13, IFNg were not displayed as they were consistently outside the assay range and there was limited material for reanalysis. The plate was analyzed using a Bio-Plex 200 System (Bio-Rad Laboratories, CA, USA) with cytokine/chemokine levels being generated automatically from standard curves using the Bio-Plex Manager software (v.4.1.1 Bio-Rad). In cases where the analyte was detected, but below the limit of detection, ½ the LOD was used for analysis. Results were normalized to total protein as determined with the Qubit Protein Assay Kit and Qubit 2.0 Flourometer (Life Technologies, CA, USA). Paired t-tests were used to make comparisons.

## Supporting Information

Figure S1 **Study design.** The study was designed to have two treatment periods separated by a washout period with a wash-in period and follow up period. Information collected at each visit is outlined in the bottom table. Visit 9 was only necessary if the probiotics were detected by culture methods.
(PDF)

Figure S2 **Heat map of probiotic OTUs as compared to the remainder of the microbiota.** Each row represents a participants sample at a given time point. The samples are labeled in the following format V(Visit#)_(Participant Identifier).
(PDF)

Figure S3 **PCoA of weighted UniFrac distances with overlaid genera.** All samples were plotted and colored by the individual of origin, again showing significant clustering by individual. Three broad groups can be determined (i) associated with *Lactobacillus*, (ii) associated with *Gardnerella* and *Atopobium* and (iii) a diverse microbiota including *Prevotella* and *Veillonella*.
(PDF)

Figure S4 **Heat map of all detected metabolites across all samples.**
(PDF)

Figure S5 **Correlation between lactate abundance and percent lactate producers.** Each dot represents a different sample and each color a different individual. The coefficient of correlation was 0.43 (*p* = 9.6×10^−6^).
(PDF)

Figure S6 **Principle component analysis (PCA) of metabolites in vaginal fluid before and after probiotic (A) or placebo (B) intervention.** Each point represents a different sample. Distribution of samples is based on metabolites alone, where the distance between samples represents how similar the metabolomes of those samples are.
(PDF)

Figure S7 **Cytokine levels across groups following before and after probiotic and placebo adjusted by total protein in the sample.** (A)IL-1β, (B) IL-4, (C) IL-5, (D) IL-6, (E) GM-CSF, (F) TNFα.
(PDF)

Figure S8 **Ingenuity Pathway Analysis of inflammatory network altered by probiotic administration.** Genes differentially expressed (red = up-regulated, green = down-regulated) by probiotic treatment were overlaid over a protein interaction network showing TLR2 and IL18 as central nodes in an inflammatory network.
(PDF)

Table S1 **Taxonomic assignments of OTUs.** The OTU number, the assigned taxonomy and the OTU seed sequence are presented.
(XLSX)

Table S2 **Filtered gene lists of probes differentially expressed before and after probiotic (A) and placebo (B) administration.** Probe sets presented were differentially expressed ≥2-fold with a raw p-value<0.05. Two complete sample sets (before and after intervention from the same individual, 4 arrays) were obtained from the probiotic intervention and 3 sets from the placebo intervention (6 arrays).
(XLSX)

Table S3 **Nucleotide barcodes for participant sample 16S rRNA sequencing.**
(XLSX)

Protocol S1 **Study protocol.**
(PDF)

Checklist S1 **CONSORT checklist.**
(DOC)
