# Supplementary figures and images for: A Systems Biology Approach Investigating the Effect of Probiotics on the Vaginal Microbiome and Host Responses in a Double Blind, Placebo-Controlled Clinical Trial of Post-Menopausal Women
Source: PLoS One. 2014 Aug 15;9(8):e104511. doi: 10.1371/journal.pone.0104511 (PMC4134203; doi:10.1371/journal.pone.0104511)

### STUDY VISIT SCHEDULE

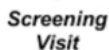[illegible]

Supplement: Figure S1 — Study design. The study was designed to have two treatment periods separated by a washout period with a wash-in period and follow up period. Information collected at each visit is outlined in the bottom table. Visit 9 was only necessary if the probiotics were detected by culture methods. (PDF) [file pone.0104511.s001.pdf]

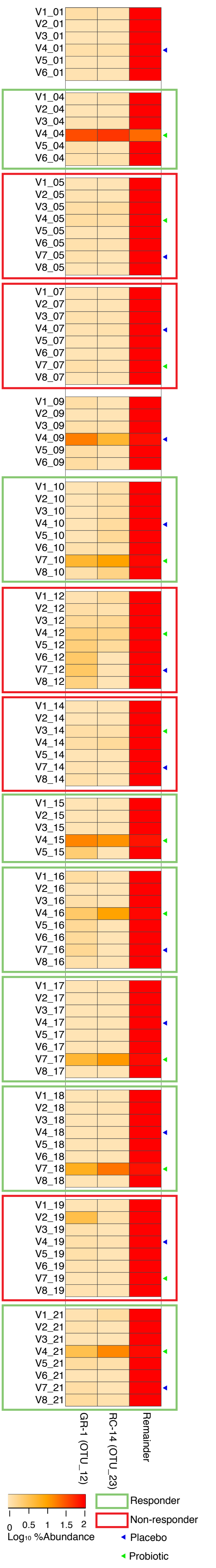

Supplement: Figure S2 — Heat map of probiotic OTUs as compared to the remainder of the microbiota. Each row represents a participants sample at a given time point. The samples are labeled in the following format V(Visit#)_(Participant Identifier). (PDF) [file pone.0104511.s002.pdf]

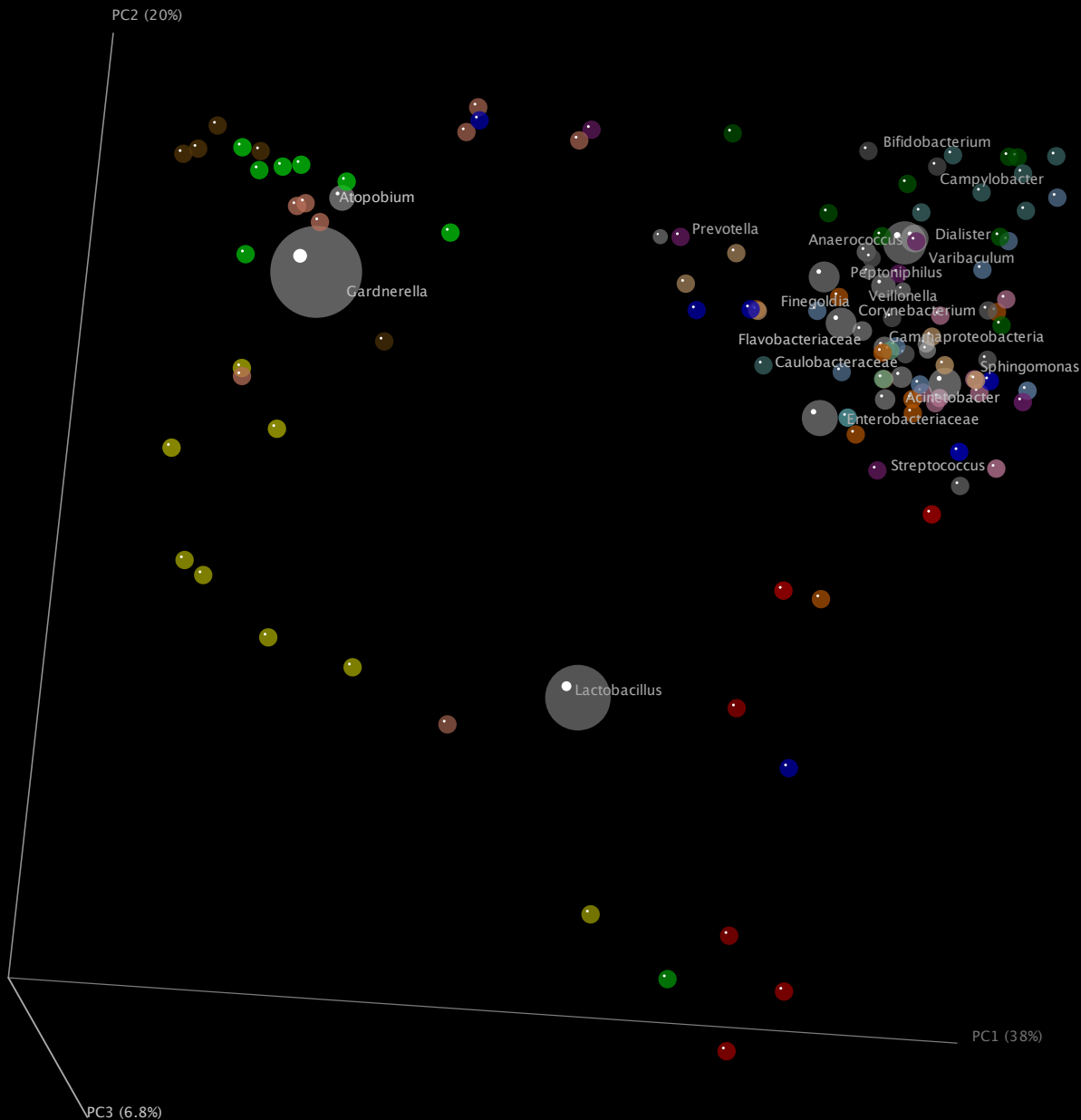

Supplement: Figure S3 — PCoA of weighted UniFrac distances with overlaid genera. All samples were plotted and colored by the individual of origin, again showing significant clustering by individual. Three broad groups can be determined (i) associated with Lactobacillus, (ii) associated with Gardnerella and Atopobium and (iii) a diverse microbiota including Prevotella and Veillonella. (PDF) [file pone.0104511.s003.pdf]

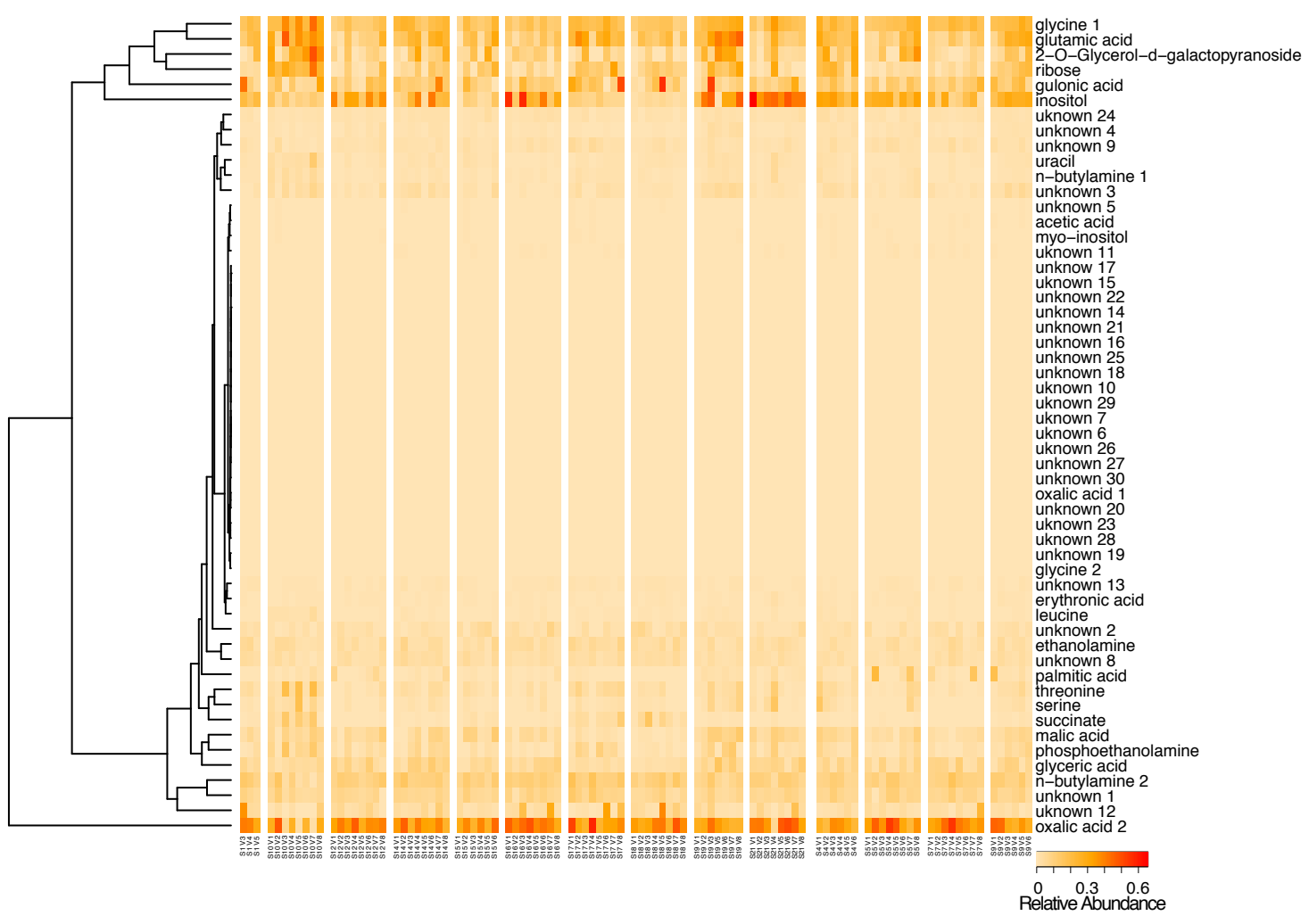

Supplement: Figure S4 — Heat map of all detected metabolites across all samples. (PDF) [file pone.0104511.s004.pdf]

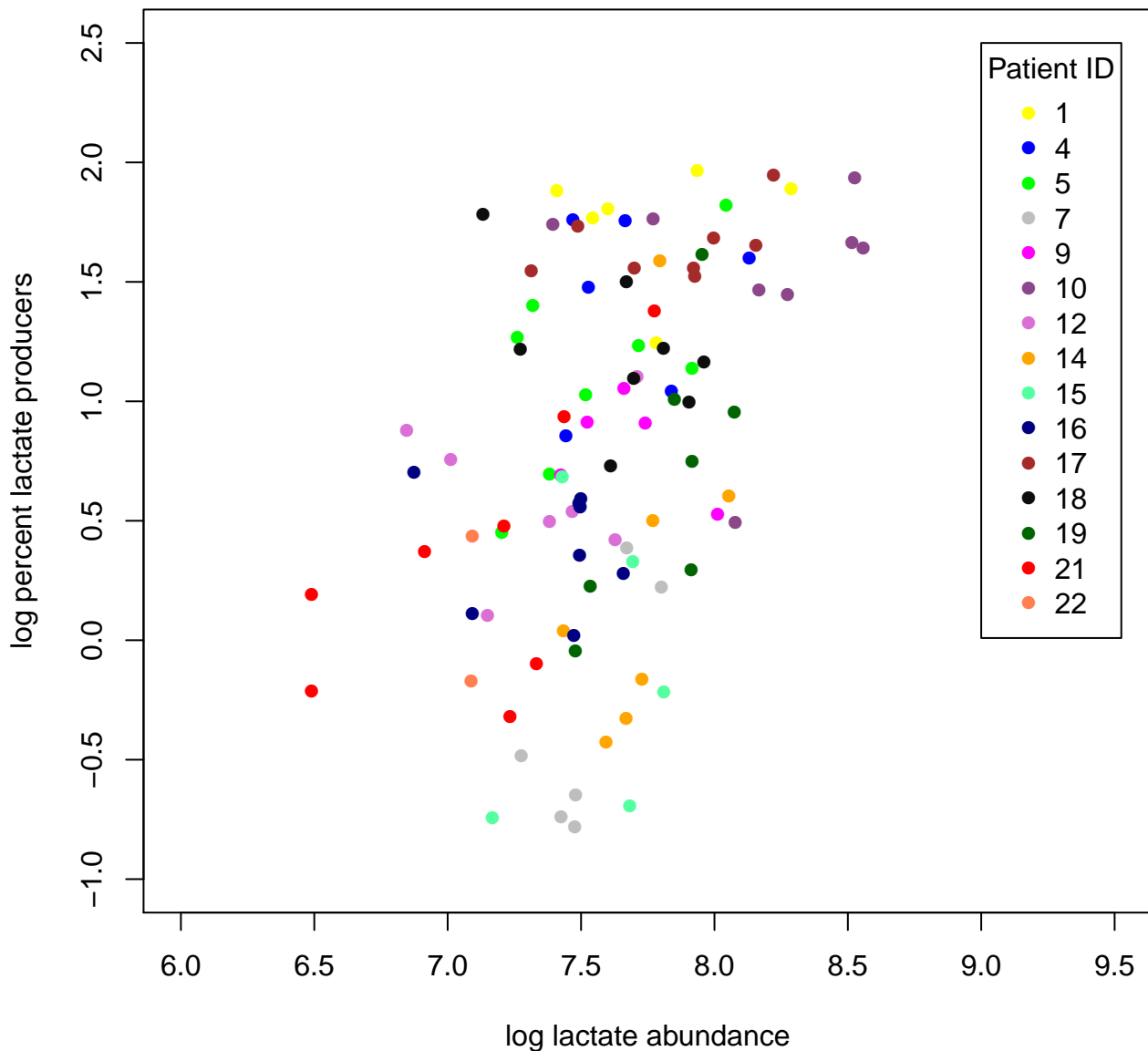

Supplement: Figure S5 — Correlation between lactate abundance and percent lactate producers. Each dot represents a different sample and each color a different individual. The coefficient of correlation was 0.43 (p = 9.6×10^−6^). (PDF) [file pone.0104511.s005.pdf]

 pro b  
 pro a

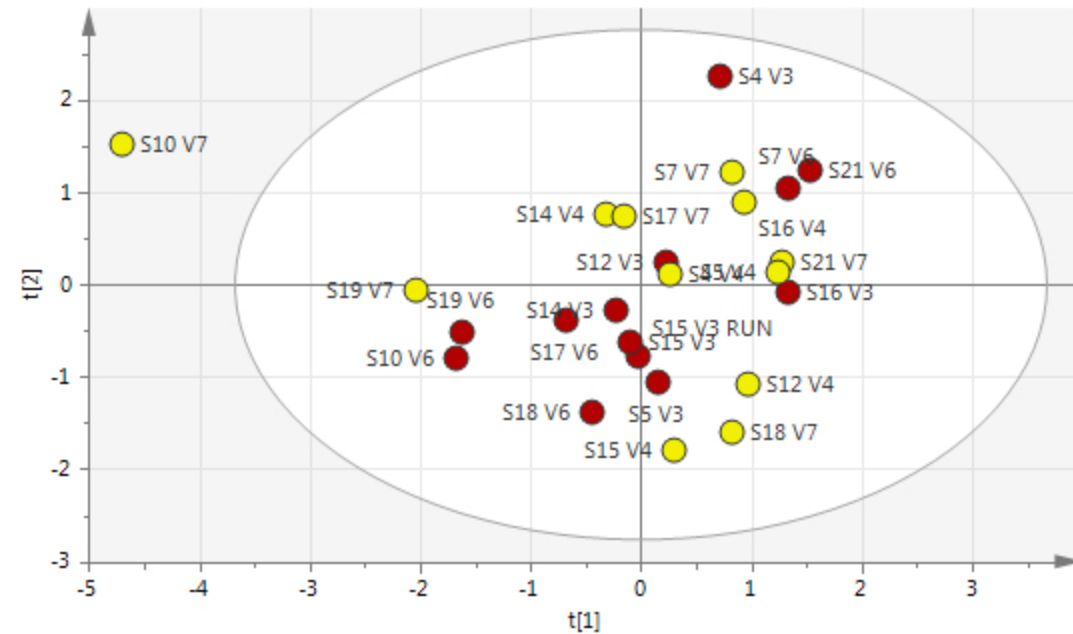

```
1] = 0.378      R2X[2] = 0.213      Ellipse: Hotelling's T2 (95%) .] = 0.316
```

SIMCA 13.0 - 9/23/2013 5:29:35 PM (UTC-4)

**B.**

 plac b  
 plac a

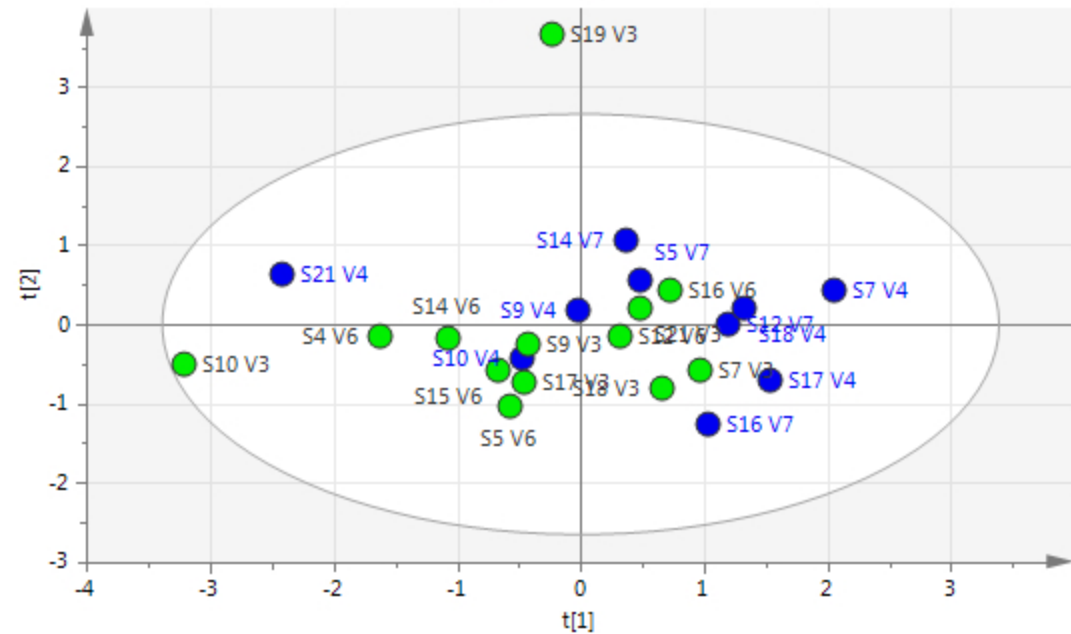

R2X[2] = 0.195      Ellipse: Hotelling's T2 (95%)

SIMCA 13.0 - 9/23/2013 5:36:08 PM (UTC-4)

Supplement: Figure S6 — Principle component analysis (PCA) of metabolites in vaginal fluid before and after probiotic (A) or placebo (B) intervention. Each point represents a different sample. Distribution of samples is based on metabolites alone, where the distance between samples represents how similar the metabolomes of those samples are. (PDF) [file pone.0104511.s006.pdf]

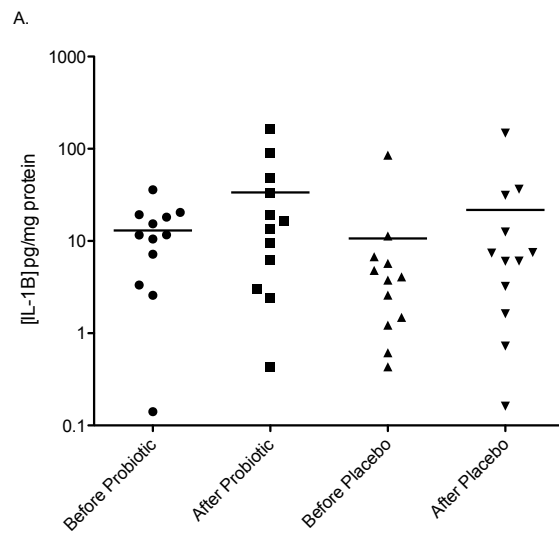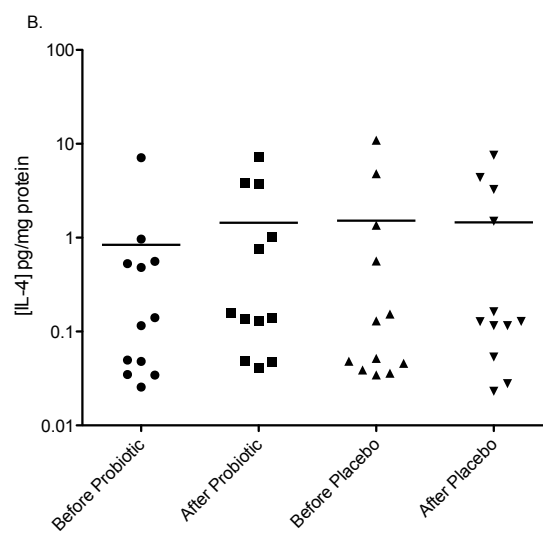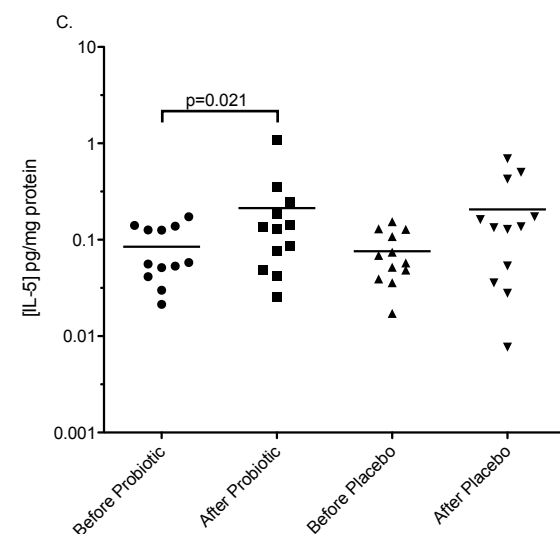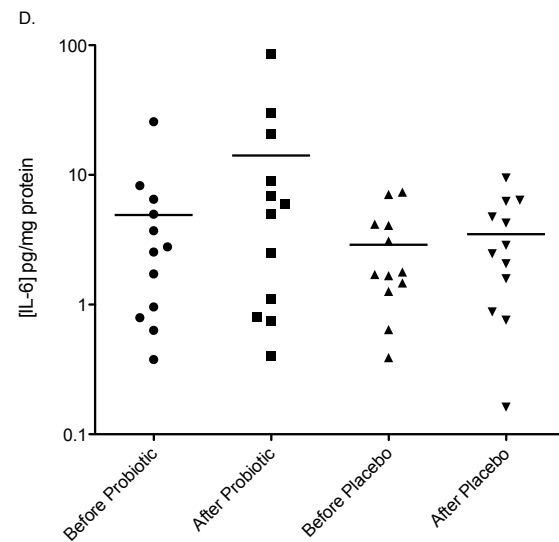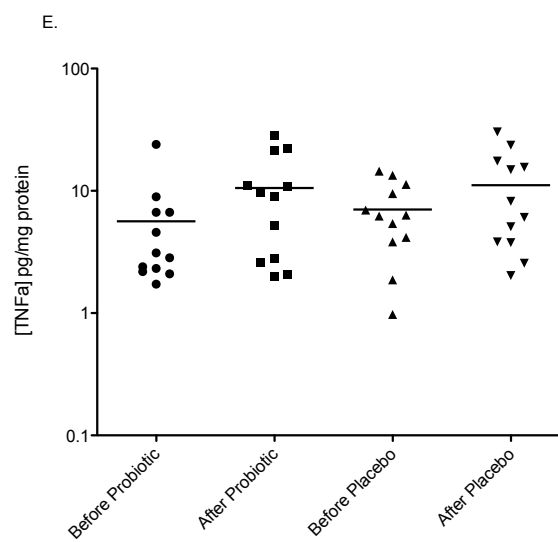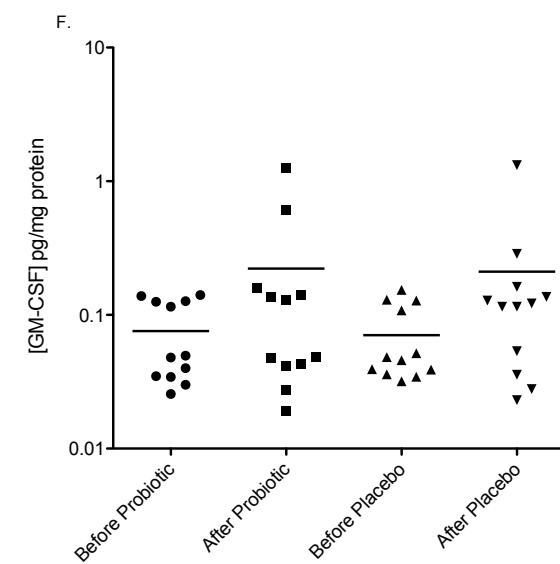

Supplement: Figure S7 — Cytokine levels across groups following before and after probiotic and placebo adjusted by total protein in the sample. (A)IL-1β, (B) IL-4, (C) IL-5, (D) IL-6, (E) GM-CSF, (F) TNFα. (PDF) [file pone.0104511.s007.pdf]

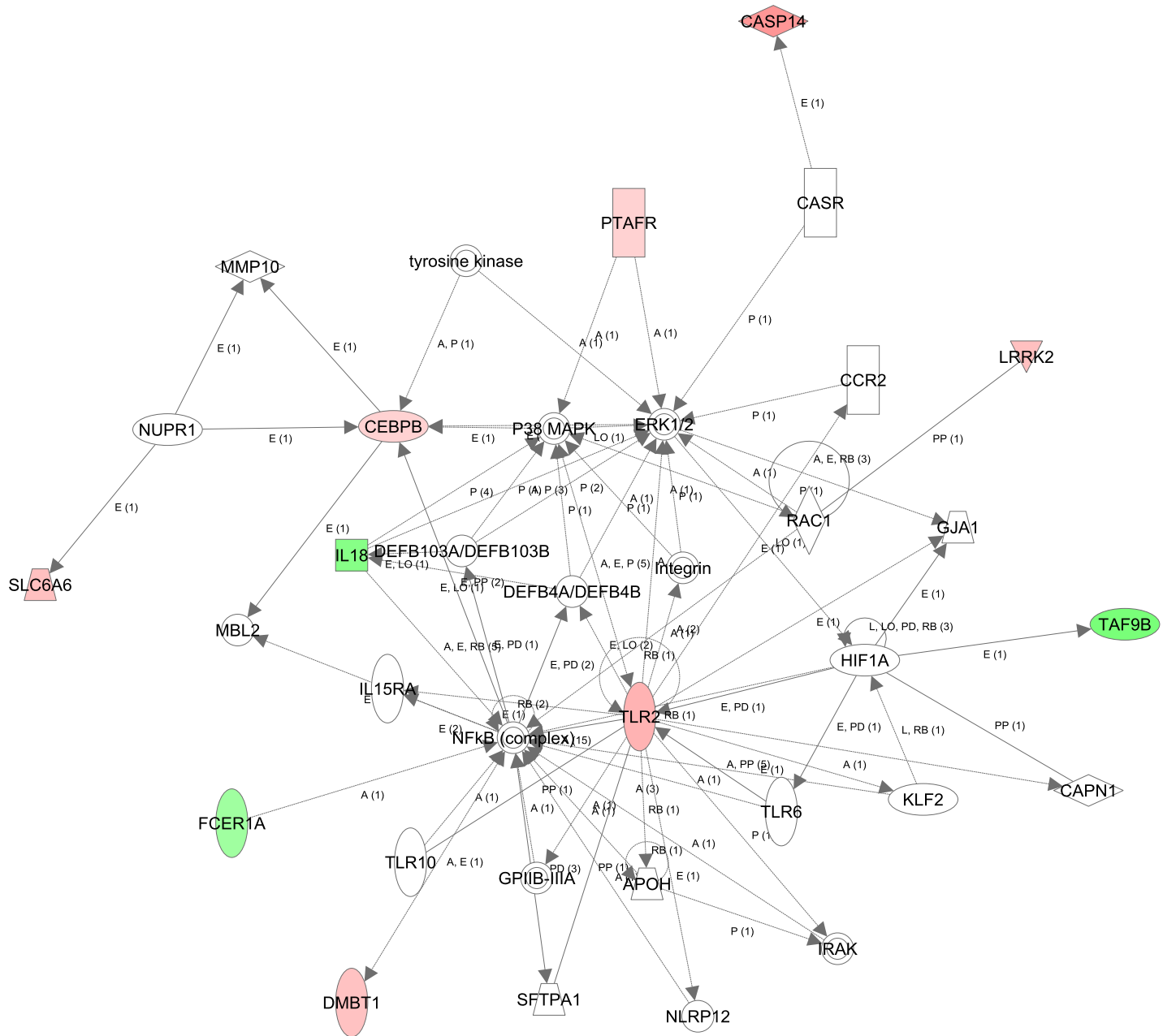

Supplement: Figure S8 — Ingenuity Pathway Analysis of inflammatory network altered by probiotic administration. Genes differentially expressed (red = up-regulated, green = down-regulated) by probiotic treatment were overlaid over a protein interaction network showing TLR2 and IL18 as central nodes in an inflammatory network. (PDF) [file pone.0104511.s008.pdf]
