# Supplementary material for: A Systems Biology Approach Investigating the Effect of Probiotics on the Vaginal Microbiome and Host Responses in a Double Blind, Placebo-Controlled Clinical Trial of Post-Menopausal Women
Source: PLoS One. 2014 Aug 15;9(8):e104511. doi: 10.1371/journal.pone.0104511 (PMC4134203; doi:10.1371/journal.pone.0104511)
Supplement: Protocol S1 — Study protocol. (PDF) [file pone.0104511.s012.pdf]

PROTOCOL

A Single-Center Exploratory Study to Evaluate the Effects of the Probiotics *L. rhamnosus* GR-1® and *L. reuteri* RC-14® on Subjects with Intermediate Nugent Scores

Protocol 500-10-0002

Including Amendment 2

Version Date: July 17,, 2012

Sponsor

Kimberly-Clark Corporation
1400 Holcomb Bridge Road
Roswell, GA 30076 USA

Protocol Signature Page

Protocol 500-10-0002 entitled A Single-Center Exploratory Study to Evaluate the Effects of the Probiotics *L. rhamnosus* GR-1® and *L. reuteri* RC-14® on Subjects with Intermediate Nugent Scores.

Lung Fai Wong, M. S.<br>*Biostatistician*	Date
Shu-Ping Yang, M. D.<br>*Associate Medical Director Kimberly-Clark*	Date

Qualified Investigator:

I have read this protocol and agree to conduct this study in accordance with the protocol and principles of Good Clinical Practice as stated in the ICH GCP Guidelines, Declaration of Helsinki and local regulations.

Qualified Investigator's Signature	Date
NAME (print)	

TABLE OF CONTENTS

1 STUDY SYNOPSIS 5
2 STUDY VISIT SCHEDULE 10
3 LIST OF ABBREVIATIONS 12
4 GENERAL INFORMATION 13
4.1 Sponsor and Contract Research Organization 13
4.2 Investigational Sites 14
5 BACKGROUND INFORMATION 14
5.1 Disease Background 14
5.1.1 Causes 15
5.1.1.1 Microbiology of the Vagina and the Role of Estrogens 15
5.1.2 Risk Factors 15
5.1.2.1 Reproductive Tract Complications 16
5.1.2.2 Sexually Transmitted Diseases 16
5.1.2.3 Recurring Infection 16
5.1.3 Diagnosis 16
5.2 Study Rationale 17
5.3 Treatment Options 17
5.3.1 Antibiotics 17
5.3.2 Probiotics 18
5.3.2.1 Mode of Action 18
5.3.2.2 Clinical Benefits of Probiotics 18
5.4 Description of the Investigational Natural Health Product (INHP) 19
5.4.1 Safety 19
5.4.2 Contraindications 20
5.4.3 Warnings and Precautions for Use 20
5.4.4 Clinical Studies 20
5.4.5 Interactions with Medicinal Products 21
5.4.6 Overdose 21
6 STUDY DESIGN 21
6.1 Study Objectives 22
6.2 Exploratory Outcomes 22
6.3 Safety Assessments 22
7 SELECTION OF SUBJECTS 22
7.1 Inclusion and Exclusion Criteria 23
7.2 Randomization Process 24
8 STUDY PROCEDURES 24
8.1 Visit Schedules 24
8.2 How to Take Vaginal Samples 28
8.3 How to Insert Capsule 28
9 INVESTIGATIONAL NATURAL HEALTH PRODUCT 29
9.1 Supply 29
9.2 Packaging 29

9.3 INHP Capsule Formulation 29
9.4 Placebo Formulation 29
9.5 Labelling 29
9.6 Storage 30
9.7 Accountability 30
9.8 Subject Compliance 30

10 CONCOMITANT MEDICATIONS AND TREATMENTS 30

10.1 Rescue Medication for BV 30
10.2 Permitted Medications and Treatments 31
10.3 Medications and Products not Permitted During the Study 31

11 SAFETY REPORTING 31

11.1 Adverse Event 31
11.2 Serious Adverse Event 31
11.3 Reporting of Serious Adverse Events 32

12 SUBJECT WITHDRAWAL 32

13 STATISTICS 33

13.1 Statistical Methods 33
13.1.1 Analysis Populations 33
13.1.2 Efficacy Analysis 33
13.1.2.1 Nugent Score Analysis 33
13.1.3 Exploratory Analyses 34
13.1.3.1 Additional Nugent Score Analyses 34
13.1.3.2 pH Analysis 34
13.1.3.3 Symptom Analysis 34
13.1.3.4 Microbiota Analysis 34
13.1.3.5 Microarray Analysis 35
13.1.3.6 Metabolomic Analysis 35
13.1.3.7 Multiplex Immunological Analysis 35
13.1.4 Safety Analysis 35
13.2 Sample Size and Power Considerations 35

14 STUDY MANAGEMENT 35

14.1 Protocol Amendments 35
14.2 Subject Confidentiality 36
14.3 Quality Control and Quality Assurance Procedures 36
14.4 Criteria for Early Termination of the Study 36
14.5 Discontinuation of the Study 36

15 ETHICS 36

15.1 Good Clinical Practice 36
15.2 Ethics Committee 37
15.3 Informed Consent 37
15.4 Direct Access to Source Data/Documents 37
15.5 Data Handling and Record Keeping 37

16 PUBLICATION POLICY 38

17 REFERENCES 38

1 STUDY SYNOPSIS

**Name of Sponsor:**	Kimberly-Clark Corporation
**Investigational Natural Health Product (INHP):**	*L. rhamnosus* GR-1® and *L. reuteri* RC-14® capsules manufactured by Chr. Hansen
**Clinical Phase:**	Pilot Trial
**Protocol Title:**	A Single-Centre Exploratory Study to Evaluate the Effects of the Probiotics *L. rhamnosus* GR-1® and *L. reuteri* RC-14® on Subjects with Intermediate Nugent Scores
**Protocol Number:**	500-10-0002
**Investigator:**	To Be Determined
**Study Center:**	To Be Determined
**Subject Population: and Number:**	A total of 14 post-menopausal women between the ages of 40 and 80 years old will be randomized in this study.
**Study Duration:**	The study treatment period is approximately seven (7) weeks, plus a 3-month follow-up period as follows:<br>• Two (2) weeks of no treatment; followed by<br>• Two (2) treatment periods, each consisting of three (3) days of a randomized placebo or probiotic treatment plus an 18 day washout period between treatments;<br>• Follow-up 3 months after the last treatment.

**Study Objectives:**	Primary Objective:<br><br>Determine whether *L. rhamnosus* GR-1® and *L. reuteri* RC-14® delivered via capsules to the vagina of post-menopausal women over a three day course of treatment can restore and maintain a lactobacilli-dominated microbiota for one week as measured by:<br><br>• A decrease in the Nugent Scores from an intermediate score (4 - 6) at baseline to a normal score (0 - 3) following treatment.<br><br>Secondary Objectives:<br><br>Determine the effect of *L. rhamnosus* GR-1® and *L. reuteri* RC-14® on the subject's anti-microbial defences and vaginal comfort as measured by:<br><br>• Microbial ecology analysis, human genetic microarrays and multiplex immunological assessments;<br><br>• An increase in symptom relief such as itching, irritation, or general vaginal discomfort;
**Exploratory Outcomes:**	• Metagenomic study to evaluate whether there is an increased abundance of lactobacilli and decrease in pathogens with probiotic therapy;<br><br>• Metabolomic study to determine whether there are differences in the factors associated with improved health between probiotic, placebo, and no treatment conditions;<br><br>• Multiplex immunological study to evaluate the modulation of anti-microbial and/or anti-inflammatory host response factors in response to the probiotic;<br><br>• Microarray analysis to determine whether there are differences in gene regulation between probiotic, placebo, and no treatment conditions; and<br><br>• Symptom analysis to evaluate whether probiotic treatment is associated with symptom relief.
**Safety Measures:**	Safety assessment will be based on the type, frequency and severity of adverse events.
**Active Therapy:**	*L. rhamnosus* GR-1® and *L. reuteri* RC-14® capsules manufactured by Chr. Hansen
**Reference Therapy:**	Placebo Comparator capsules, identical in appearance to the active product; 98% Gelatin with no *L. rhamnosus* GR-1® and *L. reuteri* RC-14®

**Dosage, Dose Form, and Route of Administration:**	*L. rhamnosus* GR-1® and *L. reuteri* RC-14® in preparations consisting of freeze-dried bacteria capsules containing 2.5 x 10^9 CFU of each of GR-1® and RC-14® (at time of manufacture).<br><br>The INHP will be administered vaginally two (2) times daily for three (3) consecutive days.
**Rescue Medication:**	Metronidazole or Clindamycin as required to treat bacterial vaginosis (BV)
**Study Design:**	This is a prospective, randomized, double-blind crossover study.<br><br>Fourteen (14) post-menopausal women with a Nugent Score of four (4) to six (6) will be included in the study.<br><br>Subjects will undergo the following treatment phases:<br>• Two weeks of no treatment (Days 1-15); followed by<br>• Three days in which each subject randomly receives either two intravaginal placebo capsules or two intravaginal probiotic capsules (Days 16-18) per day; followed by<br>• 18 day wash-out period; followed by<br>• Three days in which each subject randomly receives either two intravaginal placebo capsules or two intravaginal probiotic capsules (Days 37-39) per day.<br><br>The capsules given on Days 37-39 will not be the same as the capsules given on Days 16-18. During one treatment period the probiotic will be given and during the other treatment period, placebo will be given.<br><br>Both the subjects and the investigator will be blinded as to placebo and probiotic treatments. Subjects will provide vaginal samples on Days 1, 5, 15, 19, 26, 36, 40, and 47. An additional sample may be taken on Day 129 if the subject is still colonized at Day 47.<br><br>A diary to track compliance and to rate general vaginal discomfort and other adverse events will be given to the subjects on Day 1, Day 15 and Day 36 to be reviewed by the study Investigator or designee at the following visit.<br><br>Subjects that remain colonized with the probiotic strains at Day 47 will be queried monthly via telephone to monitor for adverse events and have a follow-up visit on Day 129 where a vaginal swab will be taken to determine if they are still colonized by the probiotic strains.<br><br>Subjects that are not colonized with the probiotic strains at Day 47 will be queried monthly via telephone for adverse events until Day 129. No visit is required at Day 129.

Key Entry Criteria:	Inclusion Criteria:<br>1. Willing and able to read, understand, and sign the Informed Consent Form (ICF);<br>2. Post-menopausal females between the ages of 40 and 80 years old (subjects who have not had a menstrual period for the last 12 months);<br>3. Currently in a mutually monogamous sexual relationship or not sexually active;<br>4. Agree to be sexually abstinent 72 hours prior to each study visit. Also, agree to refrain from intercourse for 48 hours after treatment administration;<br>5. Agree to abstain from the use of any other intravaginal product (e.g. gels, foams, lubricants, douches, etc.) throughout the study period, from the time of screening until Day 47;<br>6. Willing and capable of following all study instructions; and<br>7. Good general health.<br>Exclusion Criteria:<br>1. Use of vaginal lubricants, or any products applied vaginally within three months prior to Visit 1 and throughout the duration of study participation;<br>2. A history or currently undergoing immunosuppressive drug therapy, chemotherapy, or radiation therapy;<br>3. A medical condition which might compromise immune system functions (such as cancer, leucopenia, HIV-positive, or organ transplant);<br>4. Antibiotics and/or antifungal medication use within the last four (4) weeks;<br>5. Oral probiotic supplement use within 3 months prior to Visit 1 and throughout the duration of the study;<br>6. Significant changes in diet during the course of the study based on self-report;<br>7. Induced menopause due to surgical or medical interventions, such as bilateral oophorectomy, hysterectomy, chemotherapy or radiation treatment;<br>8. Currently undergoing local or systemic estrogen therapy who are not willing to alter therapy during the course of the study;<br>9. A Nugent Score of 0 – 3 or greater than 6;<br>10. History of drug or alcohol abuse;

	11. Currently diagnosed with or being treated for a genital infection or urinary tract infection;<br>12. Individuals with a sexually transmitted disease (self-reported or detected by the Principal Investigator);<br>13. At enrollment, have any social or medical condition, or psychiatric illness that, in the opinion of the Investigator, would preclude provision of informed consent, make participation in the study unsafe, complicate interpretation of study outcome data, or otherwise interfere with achieving the study objectives;<br>14. Participation in a clinical trial involving an investigational product/device within the past three months; subjects who are scheduled to participate in another clinical study concurrently; and<br>15. Known intolerance or allergy to *L. rhamnosus GR-1®* or *L. reuteri RC-14®* or to any product excipients.
**Sample Size:**	A total of 14 subjects with Nugent Scores in the range of 4 – 6.
**Data Analyses:**	Efficacy endpoints include the Nugent Scores and symptoms such as itching, irritation, and general vaginal discomfort, as well as other symptoms.<br><br>The primary efficacy analysis is based on the Nugent Score, as following:<br><br>H_o: $\pi_A = \pi_P$<br><br>H_a: $\pi_A \neq \pi_P$<br><br>π_A denotes proportion of subjects used intravaginal probiotic capsules had shown improvement in Nugent Score from intermediate (4-6) to normal status (0-3)<br><br>π_P denotes proportion of subjects used intravaginal placebo capsules had shown improvement in Nugent Score from intermediate (4-6) to normal status (0-3)<br><br>McNemar's test will be utilized to test the hypothesis at 5% level of significance, 2-sided.<br><br>Exploratory endpoints include the pH analyses, symptoms of itching, burning, discharge and general vaginal discomfort, microbiota analysis, microarray analysis, and multiplex immunological analysis will also be analyzed.<br><br>For safety analysis, adverse events will be tabulated by Body System, severity, and Investigator reported relationship to the test articles.

2 STUDY VISIT SCHEDULE

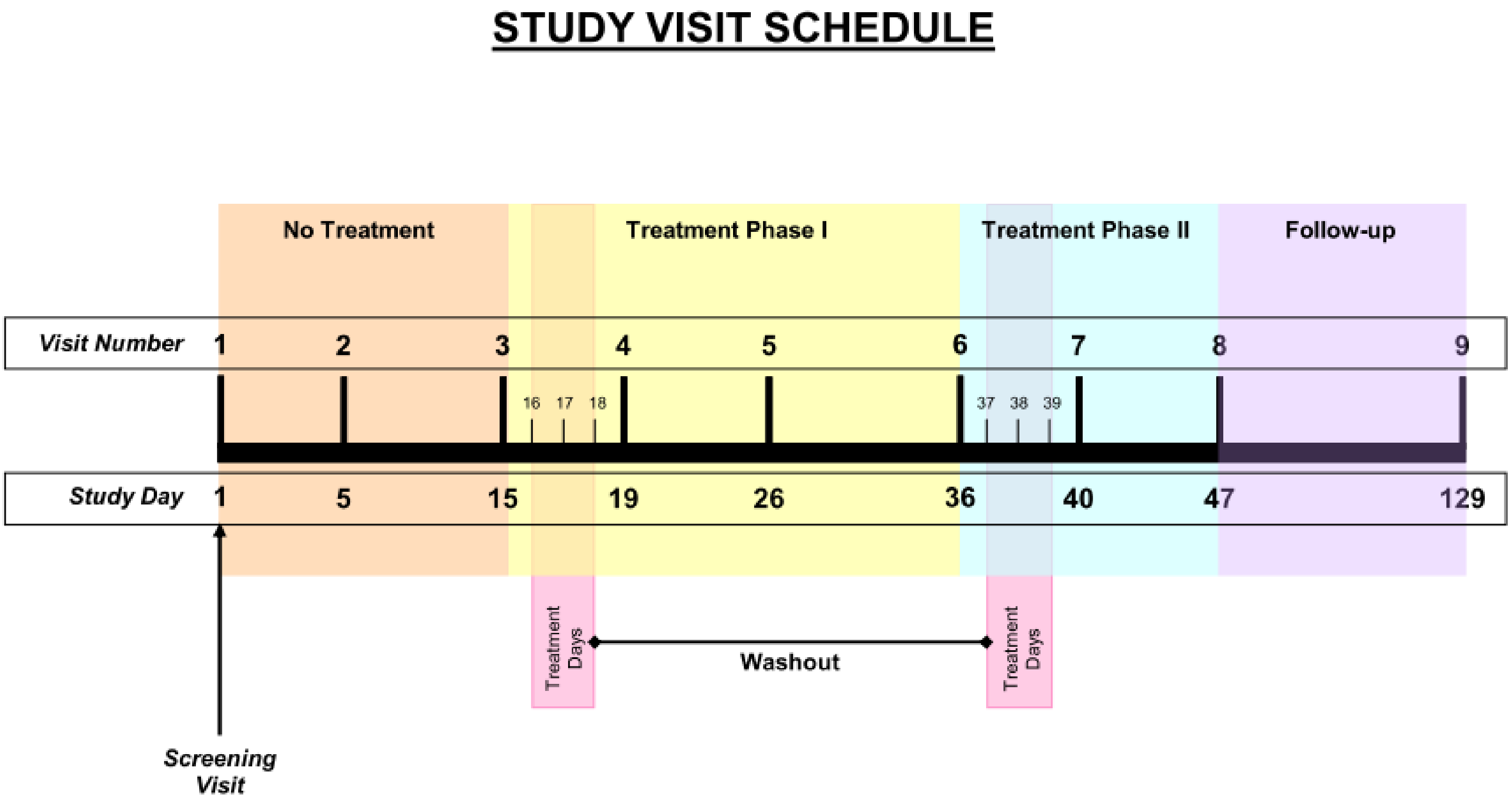

Visit No.	Visit 1	Visit 2	Visit 3	Visit 4	Visit 5	Visit 6	Visit 7	Visit 8 (End of Treatment visit)	Visit 9 Additional Follow-Up
**Study Day**	**Day 1**	**Day 5 (+1)**	**Day 15 (+/- 2)**	**Day 19 (+1)**	**Day 26 (+/- 1)**	**Day 36 (+/- 2)**	**Day 40 (+1)**	**Day 47 (+/- 2)**	**Day 129 (+/- 7)**
	**No treatment Phase**		**Treatment Phase I**			**Treatment Phase II**		**Follow-up**	
Written Informed Consent	X								
Inclusion/Exclusion Criteria	X								
Medical History	X								
Investigational NHP Dispensing			X[1]			X[2]			
Swab for Nugent Score/Microbiota Analysis	X	X	X	X	X	X	X	X	
Swab for Microbiota Analysis	X	X	X	X	X	X[3]	X	X[3]	X[3]
Cytobrush sample for Metabolomic Analysis	X	X	X	X	X	X	X	X	
Cytobrush sample for Host Gene Regulation	X	X	X	X	X	X	X	X	
Swab for Multiplex Immune Test	X	X	X	X	X	X	X	X	
Vaginal pH Measurement	X	X	X	X	X	X	X	X	
Diary Dispensing	X		X			X			
Adverse Event Monitoring		X	X	X	X	X	X	X	X
Review Diary		X	X	X	X		X	X	
Collect Diary			X		X			X	
Assess Concomitant Medications	X	X	X	X	X	X	X	X	
Randomization			X						
Vaginal Health Questionnaire	X	X	X	X	X	X	X	X	
Telephone Monitoring for Adverse Events									X[4]

KEY:
1. Subjects with a Nugent Score of 4-6 will be given six (6) capsules to be inserted vaginally twice per day for the next three (3) days. Subjects with a Nugent Score of 7-10 will be treated for BV as appropriate and discontinued from the study. Subjects with a Nugent Score of 0-3 should return in one week to have the Nugent Score reassessed.
2. Subjects with a Nugent Score of 4-6 will be given six (6) capsules to be inserted vaginally twice per day for the next three (3) days. Subjects with a Nugent Score of 7-10 will be treated for BV as appropriate and discontinued from the study. Subjects with a Nugent Score of 0-3 will be discontinued from treatment but will be followed until day 129.
3. Swabs for microbiota analysis will also be used to assess colonization by probiotic strains on Visits 6, 8, and 9. Microbiota analysis will not be performed at Visit 9.
4. Subjects who are not colonized with the probiotic strains at Visit 8 are not required to attend Visit 9, but will receive monthly AE telephone monitoring.

3 LIST OF ABBREVIATIONS

BV	Bacterial Vaginosis
CFU	Colony Forming Units
GCP	Good Clinical Practice
ICF	Informed Consent Form
ICH-GCP	International Conference on Harmonization Good Clinical Practice Guidelines
INHP	Investigational Natural Health Product
ITT	Intent-To-Treat
GC-MS	Gas Chromatography- Mass Spectrometry
GI	Gastrointestinal
GR-1®	*Lactobacillus Rhamnosus* Strain GR-1®
LC-MS	Liquid Chromatography- Mass Spectrometry
mg	Milligram
mL	Milliliter
mRNA	Messenger Ribonucleic Acid
NHP	Natural Health Product
NHPD	Natural Health Product Directorate
NMR	Nuclear Magnetic Resonance
OTU	Operational Taxonomic Units
PCR	Polymerase Chain Reaction
RC-14®	*Lactobacillus Reuteri* Strain RC-14®
QOL	Quality of Life
SAE	Serious Adverse Event
VVC	Vulvovaginal Candidiasis

4 GENERAL INFORMATION

A Single-Center Exploratory Study to Evaluate the Effects of the Probiotics *L. rhamnosus* GR-1® and *L. reuteri* RC-14® on Subjects with Intermediate Nugent Scores.

This exploratory study will be conducted in compliance with the protocol, the International Conference on Harmonization Good Clinical Practice (ICH GCP) Guidelines, the statements outlined in the Declaration of Helsinki and the applicable local regulatory requirements.

4.1 Sponsor and Contract Research Organization

Sponsor:

Kimberly-Clark Corporation
1400 Holcomb Bridge Road
Roswell, GA 30076 USA

Shu-Ping Yang, MD, PhD
Associate Medical Director
Global Clinical Affairs
Telephone: (770) 587-7267
Fax: (920) 380-8893

Contract Research Organization:

Integrated Research Inc.
1351 Sunnybrooke Blvd
Dollard-des-Ormeaux, Québec
Canada H9B 3K9

Sherene Guindi, B.Sc.
Project Manager
Telephone: (514) 683-1909
Fax: (514) 683-0121

Laboratory for Vaginal Sample Analyses:

Dr. Gregor Reid, PhD, MBA,
Human Microbiology and Probiotics, Canadian Research and Development
Centre for Probiotics
Lawson Health Research Institute
Professor,
Microbiology & Immunology, and Surgery, University of Western Ontario

Mailing address:
Lawson Health Research Institute, Rm F2-116,
268 Grosvenor Street,
London, Ontario N6A 4V2
Canada

Tel: (519) 646-6100 x 65256
Fax: (519) 646-6031
gregor@uwo.ca

4.2 Investigational Sites

One site in Canada will participate in this study.

5 BACKGROUND INFORMATION

5.1 Disease Background

Symptoms from vaginal infection represent a frequent complaint encountered by clinicians involved in women's health care and are a leading cause of patient visits[1-2]. Among the commonly found forms of vaginitis including bacterial vaginosis (BV), vulvovaginal candidiasis (VVC), and trichomoniasis, BV occurs most frequently in women of reproductive age and affects 22% to 50% of symptomatic premenopausal women in the general population[3].

Bacterial vaginosis results from an imbalance in the natural vaginal microbiota. Once considered a minor nuisance, BV has gained attention among health care providers due its increased association with a broad array of infectious morbidities of the female reproductive tract. This condition also predisposes women to increased susceptibility to sexually transmitted diseases, including human immunodeficiency virus (HIV) infections, and adverse pregnancy outcome when bacteria ascend to the upper genital tract[1,4-6].

Although BV accounts for up to 50% of cases of vaginal discharge in non-pregnant women associated infection may be asymptomatic or variably symptomatic[7-8]. According to data from a National Health and Nutrition Examination Survey, BV affects 29% of women aged 14-49 within the general population, stratified as[9]:

- Younger women (14-19 years) exhibiting lower BV frequency (23.3%); and
- Women 40-49 years showing a higher infection rate (31.3%).

Conversely, data regarding BV prevalence in post-menopausal women are limited[10]. In one northern Italian study that evaluated BV prevalence among 1486 non-pregnant women 40-79 years of age who presented for routine gynaecologic care 6% of postmenopausal women, 11% of perimenopausal women, and 9.8 % of fertile women met microbiologic criteria for a diagnosis of BV[10.]

Because many women who have BV exhibit no or minor symptoms, there is a tendency to overlook this condition. In fact, an estimated 50% of women with BV report no signs or symptoms at all i.e. are asymptomatic. Symptomatic patients who have BV normally present with an abnormal vaginal discharge exhibiting a fishy odour. The discharge is usually white or grey and may show a watery consistency. Although rare, women with BV sometimes experience burning during urination or itching around the outside of the vagina, or both[1].

Over the past two (2) decades BV has assumed greater clinical importance as epidemiologic studies unveil BV as an important risk factor for a broad range of infectious morbidities in women.

5.1.1 Causes

5.1.1.1 Microbiology of the Vagina and the Role of Estrogens

A wide range of species make up the normal microbiota in vaginal mucosa which varies between pre- and postmenopausal women. In healthy premenopausal women these are generally dominated by *Lactobacillus* species including *L. iners*, *L. crispatus*, *L. gasseri*, *L. jenesenii,* followed by *L. acidophilus, L. fermentum, L. plantarum, L. brevis, L. casei, L. vaginalis, L. delbrueckii, L. salivarius, L. reuteri,* and *L. rhamnosus*[11].

During the reproductive years, estrogen promotes the growth of lactobacilli by increasing the glycogen content in the vagina and the subsequent processing of this glycogen to glucose within vaginal epithelial cells. This cellular-manufactured glucose serves as nutrient for the lactobacilli allowing them to produce lactic acid which maintains the vaginal environment at an acidic pH (<4.7) protective against bacterial growth, particularly *Prevotella* species and *Gardnerella vaginalis*[12]. High concentrations of estrogen also increase lactobacilli adherence to vaginal epithelial cells in vitro[13].

With loss of estrogens accompanying menopause come significant local changes in the vagina, marked by thinning and paling of the vagina wall, loss of vaginal elasticity and decreases in normal vaginal secretions[12]. Declining estrogen levels is thought to lead to depletion of lactobacilli, a rise in pH, and increased vaginal colonization with enterobacteria[14].

Bacterial vaginosis results from changes in the normal local microbiota of the vagina caused by an overgrowth of one or more types of anaerobic bacteria such as *Gardnerella vaginalis*, *Morbilincus species*, *Prevotella species*, *Mycoplasma hominis* and the recently identified *Atopobium vaginae* accompanied by depletion of the protective *Lactobacillus*[15-19].

Although the methods by which lactobacilli protect against BV remain unclear they appear to involve the organism's ability to[20-25].

- Populate the vaginal epithelium and mucin layer;
- Inhibit pathogen growth and virulence expression;
- Displace pathogens; and
- Modulate host defenses.

5.1.2 Risk Factors

A number of sociodemographic risk factors for contracting BV have been proposed, but with some controversy. They include[9, 26-27]:

- Younger age;
- Being non-Hispanic black or Mexican American;
- Not completing high school;
- Living at or near the poverty level;
- Douching;
- Being sexually active;
- Using contraceptives; and

- Having multiple sex partners.

Despite the sexual risk factor, BV is considered a sexually associated condition rather than a sexually transmitted condition[1].

5.1.2.1 Reproductive Tract Complications

In non-pregnant women BV associates with a number of female reproductive tract disorders including[28-31]:

- Urinary tract infections;
- Increased infection risk after gynaecological surgery;
- Cervicitis; and
- Pelvic inflammatory disease.

In pregnant women BV may contribute to[5]:

- Spontaneous abortion;
- Preterm birth; and
- Postpartum endometritis.

5.1.2.2 Sexually Transmitted Diseases

Bacterial vaginosis has emerged as an important risk factor for sexually transmitted diseases[4,6]. One landmark cross-sectional study that reviewed data from over 37,000 women across North America, Asia, and Africa identified a greater risk of HIV infection among BV infected women. Study limitations prohibited reliable stratification of the data by sources of potential heterogeneity such as age or pregnancy[32]. Another study of 670 sexually active women, 18-30 years of age, recruited from three (3) Pittsburgh-area health care clinics also showed BV-associated increased risk of acquiring a herpes simplex virus type 2 (HSV-2) infection[4].

5.1.2.3 Recurring Infection

Recurrence of BV infection occurs in up to 30% of women within 3 months of treatment and as many as 50% of women experience a repeat episode over the long-term[33]. Factors increasing the potential for repeat episodes include[1,34-35]:

- Prior history of BV;
- Having a regular or female sex partner;
- Vaginal presence of both *Atopobium vaginae* and *Gardnerella vaginalis*; and
- Persistent bacteria causing relapse.

5.1.3 Diagnosis

Clinicians consider the Nugent scoring system, first described in 1991, as the gold standard for diagnosing BV[19]. Nugent diagnosis relies on the detection of vaginal epithelial cells infected by coccobacilli, the so-called "clue cells", upon microscopic examination of a Gram-stained smear of vaginal discharge.

The Nugent Score is calculated by assessing for the presence of:

- Large gram-positive rods (lactobacilli; Scored as 0 to 4);
- Small gram-variable rods (Scored as 0 to 4); and
- Curved gram-variable rods (Scored as 0 to 2).

The Nugent Score can range from 0 to 10 with a Score of >7 consistent with BV and a Score from 4 to 6 indicative of an intermediate microbiota. A Nugent Score from 0 to 3 indicates a normal status. Women with an intermediate Score have an approximately equal probability of developing either BV or a normal microbiota[36].

Using the "Amsel" set of clinical criteria in combination with the Nugent scoring system provides a simple and reliable tool for confirming BV presence. According to Amsel criteria BV may be defined by the following clinical signs[19,37]:

- Vaginal pH >4.5;
- Presence of adherent white discharge;
- Detection of "clue cells"; and
- Presence of an amine odour after the addition of KOH treatment.

5.2 Study Rationale

Although treatment of BV pathogens with antimicrobials yields a reasonable cure-rate, associated recurrent infections and side-effects including drug resistance remain a common problem[38]. Developing methods to promote a healthy vaginal micro-flora and symptom relief for individuals with intermediate Nugent Scores becomes an attractive target. Restoring the ecological balance in the vagina may help prevent the occurrence of BV and improve treatment options and relapse rates associated with current BV therapies. *Lactobacilli* replenishment by a vaginal probiotic may restore the normal microbial status and offer an appealing method of inhibiting BV-related pathogens, particularly in the case of postmenopausal women thought to have depleted numbers of *Lactobacilli* in their vagina.

How probiotics exert their antimicrobial effects in the vagina at the cellular level represents a series of secondary outcomes of importance. Modern experimental methods permit investigation into specific human genes that are either up-regulated or down-regulated in response to probiotic treatment within constituents of vaginal discharge. The relevant vaginal cells and microbiota may easily be recovered from vaginal specimens. Understanding the host changes affected by the probiotics at the mRNA level, and the bacterial population changes will help clarify the lactobacilli mechanism of action in the treatment of BV.

5.3 Treatment Options

5.3.1 Antibiotics

Currently antibiotics represent the treatment of choice for BV and may be administered either orally or topically as per the table below[39-41]. Despite wide-spread use of the three commonly prescribed antibiotics for the treatment of BV, these agents are not well-tolerated due to a variety of associated side-effects including gastrointestinal upset, alcohol intolerance, metallic taste, and infrequently neurological and/or hematological adverse reactions.

Table 1: Therapy for bacterial vaginosis	
**Oral**	
Metronidazole (sanofi-aventis Canada)	500 mg twice daily for 7 days
Tinidazole[a]	1 g daily for 5 days
	2 g daily for 2 days
**Topical**	
Clindamycin (Pfizer Canada)	2% cream, 5 g daily for 7 days

[a]Approved in the US, not yet available in Canada

In addition, cautionary warnings against use of metronidazole or tinidazole in patients on warfarin or lithium have been issued[40-41]. Clindamycin use may be associated with *in vitro* antimicrobial resistance[28]. All three (3) antibiotics used for the treatment of BV appear to provide equivalent efficacy and are distinguished from each other based on cost and mode of administration[1]. Recent clinical evidence supports BV subgroup specific variations in response to metronidazole and clindamycin antibiotic therapies[1,42].

5.3.2 Probiotics

Probiotics are live micro-organisms which, when administered in adequate amounts, confer a health benefit on the host. The best studied probiotics to date are lactic acid producing bacteria, particularly *Lactobacillus species* and *Bifidobacterium species*[43].

5.3.2.1 Mode of Action

Although clinical evidence supporting lactobacilli's ability to displace pathogens in the vagina exists[44], the mechanism by which lactobacilli-based probiotics confer their protective effects is not fully understood. Results from in vitro studies however, point to a complex process involving immunological and cellular processes that include[20,45-49]:

- Biosurfactant inhibition of pathogens;
- Lactobacilli coaggregation with pathogenic bacteria;
- Bacteria–bacteria signaling;
- Immune modulation;
- Increase in granulocyte-colony stimulating factor (G-CSF) cytokine;
- Defensins and IL-8 neutrophil recruitment; and
- Disruption of Gardnerella and Atopobium biofilms.

5.3.2.2 Clinical Benefits of Probiotics

A number of clinical trials have investigated the use of probiotics or candidate probiotics for the treatment of BV. These are summarized in a recent review by MacPhee et al[38]. Cure rates in women receiving probiotics delivered intravaginally typically exceeded 50% and reached as high as 100% in one trial[38,50,51]. Rossi et al. assessed qualitatively the effectiveness of *L. rhamnosus* at normalizing vaginal pH and improving BV symptoms[52]. Participants reported decreases in itching, vaginal discharge and burning at the 12-month and 24-month follow-up visits.

Probiotics administered for urogenital health in general, have been well-tolerated[11]. Side-effects from probiotic treatment, although rare, have occurred and included bacteria in the blood,

inflammation in the lining of the heart and localized infections that were possibly linked to heavy consumption of dairy products[53].

Lactobacilli, including the species *L. rhamnosus* and *L. reuteri* are normal inhabitants of the gastrointestinal (GI) and urogenital tracts, and as such, *Lactobacillus rhamnosus* GR-1® (GR-1®) and *Lactobacillus reuteri* RC-14® (RC-14®) were recovered from the healthy urethra and vagina, respectively. The intended indication for this product is to maintain a healthy vaginal microbiota and thereby prevent vaginal infections such as BV and VVC. Studies investigating use of GR-1® and RC-14® in prevention of BV in women without symptoms of BV have suggested that, GR-1® and RC-14® can prevent development of BV (as defined by high or intermediate Nugent Scores) and reduce Nugent Scores[54].

Studies investigating transient colonization of GR-1® and RC-14® in the GI and vaginal tracts have shown that detection of GR-1® and RC-14® (after oral and after vaginal application) declines gradually with time and that the strains cannot be detected 30 days after cessation of oral administration[54].

5.4 Description of the Investigational Natural Health Product (INHP)

The INHP capsule contains two probiotics: GR-1® and RC-14®. The product contains freeze-dried bacteria and excipients in a gelatin capsule. This composition is formulated to contain a minimum of 5 billion (5.0×10^9) Colony Forming Units (CFU) per capsule, including equal numbers of the two strains; i.e. 2.5×10^9 CFU of each of GR-1® and RC-14® at the time of manufacture.

L. rhamnosus is a natural organism found in the healthy intestine, vagina and distal urethra[55]. The species is often used in the preparation of food (e.g. cheese production and milk fermentation).

L. reuteri is found in the intestinal tract of a number of mammalian animals, including humans as well as in a variety of fermented foods. Indeed, *L. reuteri* has been isolated in living form from every part of the digestive tract – the oral cavity, the stomach, small intestine, and colon, as well as from stool samples and from vagina.

GR-1® and RC-14® are gram positive, catalase-negative, rod-shaped bacteria. The strains are non-motile, non-hemolytic and do not form endospores, have an optimum temperature of 37 °C and belong to a genus of facultative anaerobic bacteria.

The capsule product with GR-1® and RC-14® was approved as a Natural Health Product by the Canadian authorities in 2009 and as a New Dietary Ingredient by the US Food and Drug Administration (FDA) in 2008. The International Dairy Federation has published a list of cultures (including *L. rhamnosus* and L. *reuteri*) with a documented history of safe use by the food industry and accepted by the members of the International Dairy Federation[56].

5.4.1 Safety

Many years of experience with *L. rhamnosus* and *L. reuteri* has shown their safety as an ingredient in food and food supplements and this has been tested in numerous clinical studies in doses up to at least 10×10^9 CFU/day without giving rise to any safety concerns. Use of *L rhamnosus* in general and GR-1® and RC-14® specifically is concluded to be safe in pregnant and lactating women[54].

5.4.2 Contraindications

GR-1® and RC-14® should not be given to subjects with intolerance towards lactobacilli or any of the excipients used in the formulation.

5.4.3 Warnings and Precautions for Use

The product may contain trace of milk. The product should be used with caution in severely ill and/or immunocompromised patients. Medical help should be sought in case of unfamiliar vaginal odour, discharge or pain.

5.4.4 Clinical Studies

GR-1® and RC-14® as an oral capsule product or in a vaginal capsule formulation have been used in several clinical trials for various different indications. Although none of the studies were designed as safety trials, the overall reporting of AEs was low[57].

One study randomized 125 Black-African women with Nugent Score 7-10 to either GR-1® and RC-14® or placebo for 30 days[58]. All subjects were given metronidazole 1 g daily for the first seven days of the intervention. Forty-three of 49 subjects (88%) in the probiotic group had normal Nugent Scores and negative sialidase results (an enzymatic test used by some to diagnose BV) compared to 23 of 57 subjects (40%) in the antibiotic/placebo group ($p>0.001$). This study was subject to a risk of attrition bias as 16 out of 65 in the antibiotic/probiotic group were lost to follow-up compared to 3 out of 60 in the placebo/antibiotic group with no intention-to-treat analysis performed.

Sixty-four Brazilian women with BV (Nugent Score 7-10) were randomized to either GR-1® and RC-14® or placebo for 28 days[59]. All women were given a single dose of tinidazole 2 g. Cure was defined as relief of BV signs according to Amsel's criteria and change in Nugent Score. After 28 days of intervention, 4 out of 32 (12.5%) in the probiotic/antibiotic groups had positive Amsel test, compared to 16 out of 32 (50%) in the placebo/antibiotic group. Moreover, 75% of the probiotic/antibiotic group had a Nugent Score 0-3 compared to 34.4% in the placebo/antibiotic group ($p=0.011$) at the end of the treatment period.

Hummelen and co-workers investigated long-term efficacy by randomizing 65 Black-African women who were HIV positive and had Nugent Score 4-10 to receive metronidazole 800 mg daily for 10 days and either GR-1® and RC-14® or placebo for 25 weeks[60]. Primary efficacy endpoint was BV cure rate after two weeks treatment in women with Nugent Scores 7-10 at baseline. Secondary endpoints were recurrence rate of BV at 25 weeks, and prevention of BV in women with Nugent Scores 4-7 (asymptomatic BV) at baseline. For the analysis of data, subjects were grouped according to Nugent Scores. After two weeks, women who had BV (Nugent Score 7-10) at baseline 40% in the probiotic/antibiotic group were cured compared to 55% in the placebo/antibiotic group ($p=0.3$). After 15 weeks there was a trend for higher prevalence of normal Nugent Score among the probiotic/antibiotic group (9 out of 17-53%) compared to the placebo/antibiotic group (5 out of 20=25%) ($p=0.08$), however, no statistical significant difference was found between the two groups at any time-point. The study suffered from risk of attrition bias, since 14 out of 65 randomized subjects did not complete the study.

In ten (10) clinical trials where a total of 341 healthy subjects were enrolled, four (4) cases of headache were reported and 2 cases of increased appetite[57]. In a study by Anukam et al, 39 subjects received oral capsules of GR-1® and RC-14®, of which a few subjects reported a

single episode of headache and nausea[57]. The most commonly reported AE was headache, which was evaluated to be unrelated to ingestion of the study product.

In three (3) clinical trials investigating the effect of GR-1® and RC-14® in women without clinical symptoms of BV, including one study in women with HIV, 71 subjects were enrolled, seven (7) subjects reported GI AEs and one (1) case of an unspecified vaginal AE. All events were rated mild and the rate and incidence were similar to the placebo group[60].

5.4.5 Interactions with Medicinal Products

Lactobacilli are sensitive to numerous antimicrobial products, and concomitant therapy (local and/or systemic) can lead to a decrease in efficacy. Lactobacilli are generally resistant to nitroimidazole antibiotics (such as metronidazole), which is commonly used to treat BV[61]. There are no known interactions between probiotics and other medicinal products.

5.4.6 Overdose

No overdose with GR-1® and RC-14® has been reported. The human gut contains approximately 1 x 10^{12} CFU of bacteria/gram faeces and thus an overgrowth based on capsules containing 1-5 x 10^{9} seems an unlikely scenario. High doses of a *Lactobacillus* product (1 x 10^{11} CFU per day) have been reported to be well-tolerated[62]. This corresponds to 50-100 capsules of this investigational product.

6 STUDY DESIGN

The study population will consist of post-menopausal subjects presenting with intermediate Nugent Scores (4-6). The study duration is 47 days with a final follow-up at 129 days. A total of fourteen (14) subjects will be randomized into this exploratory study.

Subjects will undergo the following treatment phases:

- Two weeks of no treatment (Days 1-15); followed by
- Three days in which each subject randomly receives either two intravaginal placebo capsules or two intravaginal probiotic capsules (Days 16-18) each day; followed by
- 18 day wash-out period; followed by
- Three days in which each subject randomly receives either two intravaginal placebo capsules or two intravaginal probiotic capsules (Days 37-39) each day; and
- Follow-up approximately 90 days after last treatment.

The capsules given on Days 37-39 will not be the same as the capsules given on Days 16-18. During one treatment period the probiotic will be given and during the other treatment period, placebo will be given. Not the subject nor the study staff will know which capsules contain the probiotics.

INHP (placebo or probiotic) consists of capsules to be administered vaginally on study treatment days; once in the morning and once in the evening approximately 12 hours apart.

Following the first two weeks of no treatment, subjects will be given six (6) placebo or probiotic capsules, on Day 15, to be administered vaginally twice daily for 3 consecutive days. Panty liners will be given to use on treatment days. On Day 36, subjects will again be given six (6) placebo or probiotic treatment capsules to be administered vaginally twice daily for

3 consecutive days in the same manner. A diary to track compliance and to rate general vaginal discomfort and record any other adverse events will be given to the patients and reviewed by the study Investigator or designee at the following visit. Subjects that remain colonized with the probiotic strains at Day 47 will return for a follow-up visit on Day 129. During the follow-up phase, all subjects who received treatment will be queried monthly via telephone regarding adverse events on a monthly basis until Day 129. See Section 8 for visit details.

6.1 Study Objectives

Primary Objectives:

The objective of this study is to determine whether *L. rhamnosus* GR-1® and *L. reuteri* RC-14® delivered via capsules to the vagina of post-menopausal women over a three day course of treatment can restore and maintain a lactobacilli-dominated microbiota for one week as measured by:

- A decrease in the Nugent Scores from an intermediate score (4 - 6) at baseline to a normal score (0 - 3) following treatment.

Secondary Objectives:

Determine the effect of *L. rhamnosus* GR-1® and *L. reuteri* RC-14® on the subjects' anti-microbial defences and vaginal comfort as measured by:

- Microbial ecology analysis, human genetic microarrays and multiplex immunological assessments; and
- An increase in symptom relief such as itching, irritation, or general vaginal discomfort.

6.2 Exploratory Outcomes

- Metagenomic study to evaluate whether there is an increased abundance of lactobacilli and decrease in pathogens with probiotic therapy;
- Metabolomic study to determine whether there are differences in the factors associated with improved health between probiotic, placebo, and no treatment conditions;
- Multiplex immunological study to evaluate the modulation of an anti-microbial and/or anti-inflammatory in the host in response to the probiotic;
- Microarray analysis to determine whether there are differences in gene regulation between probiotic, placebo, and no treatment conditions; and
- Symptom analysis to evaluate whether probiotic treatment is associated with symptom relief.

6.3 Safety Assessments

Safety will be assessed through monitoring of physical examination changes and review of adverse events recorded in the subject diary or at the study visits or by telephone.

7 SELECTION OF SUBJECTS

All entry criteria must be met in order for the subject to be entered into the study. Exemptions or waivers to the study criteria will not be granted.

7.1 Inclusion and Exclusion Criteria

Inclusion Criteria:

1. Willing and able to read, understand, and sign the ICF;
2. Post-menopausal female between the ages of 40-80 years old (subject has not had a menstrual period for the past 12 months);
3. Currently in a mutually monogamous sexual relationship or not sexually active;
4. Agree to be sexually abstinent 72 hours prior to each study visit. Also agree to refrain from intercourse for 48 hours after treatment administration;
5. Agree to abstain from the use of any other intravaginal product (i.e. gels, foams, lubricants, douches, etc.) throughout the study period, from the time of screening until Day 47;
6. Willing and capable of following all study instructions; and
7. Good general health.

Exclusion Criteria:

1. Use of vaginal lubricants, or any products applied vaginally within three months prior to *Visit* 1 and throughout the duration of study participation;
2. A history or currently undergoing immunosuppressive drug therapy, chemotherapy, or radiation therapy;
3. A medical condition which might compromise their immune system functions (such as cancer, leucopenia, HIV-positive, or organ transplant);
4. Antibiotics and/or antifungal medication use within the last four (4) weeks;
5. Oral probiotic supplement use within 3 months prior to Visit 1 and throughout the duration of the study;
6. Significant changes in diet during the course of the study.
7. Induced menopause due to surgical or medical interventions, such as bilateral oophorectomy, hysterectomy, chemotherapy and radiation treatment;
8. Currently undergoing local or systemic estrogen therapy who are not willing to alter therapy during the course of the study;
9. A Nugent Score of 0 – 3 or greater than 6;
10. History of drug or alcohol abuse;
11. Currently diagnosed with or being treated for a genital infection or urinary tract infection;
12. Individuals with a sexually transmitted disease (self-reported or detected by the Principal Investigator);
13. At enrollment, have any social or medical condition, or psychiatric illness that, in the opinion of the Investigator, would preclude provision of informed consent, make participation in the study unsafe, complicate interpretation of study outcome data, or otherwise interfere with achieving the study objectives;

14. Participation in a clinical trial involving an investigational product within the past three months; subjects who are currently scheduled to participate in another clinical study concurrently; and
15. Known intolerance or allergy to *L. rhamnosus* GR-1® and *L. reuteri* RC-14® or to any product excipient.

7.2 Randomization Process

Subjects screened in this study will be sequentially granted a three digit study number. For each study number a randomization envelope is supplied indicating the treatment assigned for both Treatment Phase I and Treatment Phase II. Treatment should be given in the order listed in the randomization envelop. The bottles are labelled as “J” and “K” from the supplier who knows the content of each bottle. Study staff and study subjects will be blinded as to study treatment being given: the probiotics or placebo.

8 STUDY PROCEDURES

8.1 Visit Schedules

Screening and Visit 1 – Day 1

At Visit 1 subjects who sign the Informed Consent Form and the Consent for Donation and Storage of Patient Samples for Research Tests form will be allocated a two digit screening number e.g. 01, 02, 03 etc. The screening number must be assigned in sequence and must not be skipped. Screening numbers may not be reassigned for any reason.

Each subject screened in the study will also be identified by three letters: the first initial of the first name, the first initial of the middle name (if the subject does not have a middle name, a dash should be inserted) and the first initial of the family name.

Before any study-related procedures are done, the subject must participate in the informed consent process and sign the Informed Consent Form.

The following assessments will be performed at Visit 1:

- Inclusion and exclusion criteria will be reviewed;
- Medical health history will be evaluated;
- A vaginal health questionnaire will be completed;
- Review concomitant medications; and
- One (1) vaginal swab will be taken to determine Nugent Score (see section 8.2(v)).

Subjects who have met the inclusion and exclusion criteria will continue in the study.

Enrolled subjects will have the following assessments performed:

- Four (4) additional vaginal samples will be taken during this visit in the following order (see section 8.2(i–iv)):
  - one swab for multiplex immunologic analysis;
  - one cytobrush sample for metabolomic analysis;
  - one cytobrush sample to evaluate host gene regulation;
  - one swab for microbiota analysis; and

- A vaginal pH measurement will be taken with a vaginal pH indicator strip, pHem Alert.

Subjects will be given a diary to rate general vaginal discomfort and record any other adverse events. An appointment will be scheduled to return in 4 days (5 days is acceptable) for Visit 2.

Subjects with an intermediate Nugent Score may or may not exhibit signs of a vaginal infection such as itching, burning during urination, and/or an abnormal or foul-smelling discharge.

Visit 2- Day 5 (+ 1 day)

The following assessments will be performed:

- Subject diaries will be reviewed and subjects queried regarding any adverse events the subject may have experienced;
- A vaginal health questionnaire will be completed;
- Five (5) vaginal samples in the following order:
    - one swab for Nugent Score/microbiota analysis;
    - one swab for multiplex immunologic analysis;
    - one cytobrush sample for metabolomic analysis;
    - one cytobrush sample to evaluate host gene regulation;
    - one swab for microbiota analysis;
- A vaginal pH measurement will be taken in the same manner as described for Visit 1;
- Review concomitant medications; and
- An appointment will be scheduled for Day 15 visit.

Treatment Phase I - Visit 3 – Day 15 (+/-2 days)

The following assessments will be performed:

- Subject diaries will be collected and reviewed, and subjects queried regarding any adverse events they may have experienced;
- A vaginal health questionnaire will be completed;
- Five (5) vaginal samples and a vaginal pH measurement will be taken in the same manner as described for Visit 2;
- Randomize subjects with Nugent Scores between four (4) and six (6) as per section 7.2;
- Subjects with a Nugent Score ranging from four (4) to six (6) will be given either six (6) placebo or six (6) probiotic capsules per the randomization and will be instructed to administer them vaginally twice daily, one in the morning and one in the evening, for the next 3 consecutive days. Written instructions for product administration will be provided. Panty liners will be supplied to use on treatment days;
- Subjects will be instructed to keep the capsules in the container and refrigerated between 4°C and 6°C until use;
- Diaries will be dispensed to track compliance and to rate any general vaginal discomfort and record any other adverse events;
- Review concomitant medications; and

- An appointment will be scheduled to return in 4 days (5 days is acceptable) for the Day 19 visit.

Subjects with a Nugent Score of 7 – 10 at Visit 3 will be treated for Bacterial Vaginosis (BV) as deemed appropriate by the Investigator and discontinued from the study. End of treatment assessments and procedures (Visit 8) should be done.

Subjects with a Nugent Score of 0 – 3 at Visit 3 will be asked to return in 7 days (+/-1 day) to have the Nugent Score reassessed. If subjects continue to have a Nugent Score of 0 – 3 after one week, they will be discontinued from the study. End of treatment assessments and procedures (Visit 8) should be done.

Visit 4 – Day 19 (+1 day)

Subjects will return to the clinic four (4) days after Visit 3. The following assessments will be performed:

- Subject diaries will be reviewed and subjects queried regarding any adverse events they may have experienced;
- A vaginal health questionnaire will be completed;
- Five (5) vaginal samples and a vaginal pH measurement will be taken in the same manner as described for Visit 2;
- Subjects' diaries will be returned to the subjects to rate general vaginal discomfort and record any other adverse events daily until their next visit;
- Review concomitant medications; and
- An appointment will be scheduled for Day 26 visit.

Visit 5 – Day 26 (+/- 1 day)

Subjects will return to the clinic seven (7) days after Visit 4. The following assessments will be performed:

- Subject diaries will be collected and reviewed and subject will be queried regarding any adverse events they may have experienced;
- A vaginal health questionnaire will be completed;
- Five (5) vaginal samples and a vaginal pH measurement will be taken in the same manner as described for Visit 2;
- Review concomitant medications; and
- An appointment will be scheduled for Day 36 visit.

Treatment Phase II - Visit 6 – Day 36 (+/-2 days)

The following assessments will be performed:

- Subjects will be queried regarding any adverse events they may have experienced;
- A vaginal health questionnaire will be completed;
- Five (5) vaginal samples and a vaginal pH measurement will be taken in the same manner as described for Visit 2;

- Subjects that have a Nugent Score of 4 – 6 will then be given either six (6) placebo or six (6) probiotic capsules per the randomization and will be instructed to administer them vaginally two (2) times daily for the next 3 consecutive days. Written instructions for product administration will be provided. Panty liners will be supplied to use on treatment days;
- Subjects will be instructed to keep the capsules in the container and refrigerated until use;
- Subjects will be given a diary to track compliance and to rate any general vaginal discomfort and record any other adverse events;
- Review concomitant medications; and
- An appointment will be scheduled to return in 4 days (5 days is acceptable) for Day 40 visit.

Subjects with a Nugent Score of 7 – 10 at Visit 6 will be treated for BV as appropriate by the Investigator and discontinued from the study. End of treatment assessments and procedures (Visit 8) should be done.

Subjects with a Nugent Score of 0 – 3 at Visit 6 will be discontinued from treatment. If these subjects are still colonized with at least one of the probiotic strains they will return for a final study visit on Day 129. All subjects discontinued from treatment will be queried via telephone regarding adverse events on a monthly basis until Day 129.

Follow-Up Phase - Visit 7 – Day 40 (+1 day)

The following assessments will be performed:

- Subject diaries will be reviewed and subjects queried regarding any adverse events they may have experienced;
- A vaginal health questionnaire will be completed;
- Five (5) vaginal samples and a vaginal pH measurement will be taken in the same manner as described for Visit 2;
- Subjects' diaries will be returned to them to rate general vaginal discomfort and record any other adverse events daily until their next visit;
- Review concomitant medications; and
- An appointment will be scheduled for Day 47 visit.

Visit 8 – Day 47 (+/-2 days)

The following assessments will be performed:

- Subject diaries will be collected and reviewed and subjects will be queried regarding any adverse events they may have experienced;
- A vaginal health questionnaire will be completed;
- Five (5) vaginal samples and a vaginal pH measurement will be taken in the same manner as described for Visit 2; and
- Review concomitant medications.

Visit 9 – Day 129 (+/-7 days) Additional Follow-Up

Subjects who are still colonized with at least one of the probiotic strains at Visit 8 (or at Visit 6 if they discontinued treatment at that time) will return for a final study visit. The following assessments will be performed:

- Subject will be queried regarding any adverse events they may have experienced; and
- One (1) vaginal swab will be taken in the same manner as the micrbiota swab described in Section 8.2. This swab will not be used to perform the microbiota analysis however; the swab will only be used to determine whether the subject continues to be colonized by the probiotic.

Follow-up will be conducted for all treated subjects (including those that were discontinued). All treated subjects will be queried via telephone regarding adverse events on a monthly basis until Day 129.

8.2 How to Take Vaginal Samples

The Vaginal pH.: Vaginal pH will be measured using the pHem-Alert indicator (Gynex, Wyoming, USA) according to the manufactures instructions.

Swabs for multiplex immunologic analysis, bacterial DNA extraction, microarray analysis, metabolmic analysis, and Nugent Score. Using a speculum the mid lateral vaginal wall is made visible, samples will be collected by the physician or nurse as follows: the swab or cytobrush will be gently pressed against the left mid lateral wall and rolled a total of two full times by twisting the wrist, then the same swabs/cytobrush will sample the right lateral wall.

(i) The first Dacron swab will be streaked across a glass slide for **Nugent scoring** by placing 8µl of sterile water onto a slide and gently rolling the swab on the slide. The swab will then be placed in a dry tube and placed at 4°C (±2°C). This swab will be used in conjunction with the final swab (see (v) below) for bacterial **DNA extraction**.

(ii) The second Dacron swab will be cut and placed in a 1.5 mL tube dry, and placed immediately in a -20°C (±2°C) freezer for **Multiplex cytokine and chemokine analysis**.

(iii) The third specimen will be collected with a cytobrush (Cytobrush plus GT, Cooper Surgical Inc. USA). After sampling both lateral walls, the cytobrush will be placed in a dry tube and placed at 4°C (±2°C) for **metabolomics analysis**.

(iv) The fourth specimen will also be collected with a cytobrush. After sampling both lateral walls, the cytobrush will be swirled around in a 1.5 ml RNase-free microtube containing RNA stabilization solution. The samples will be placed immediately in a refrigerator at 4°C (±2°C) for **microarray processing.** These samples can be retained in the refrigerator for 24 hours.

(v) A final swab (Dacron) will be collected and placed in a dry tube and placed at 4°C (±2°C), or on ice, and transported to the laboratory for plating on MRS agar and semi-quantitative analysis of Lactobacilli and **bacterial DNA extraction**.

8.3 How to Insert Capsule

In order to properly administer the INHP, the study subject should be instructed to wash their hands carefully with soap and water before commencing. Then using their index finger, the

capsule should be gently inserted into the vagina as far as comfortable and to then lie in a horizontal position for 15 minutes. Wash hands with soap and water when completed.

9 INVESTIGATIONAL NATURAL HEALTH PRODUCT

9.1 Supply

The INHP will be supplied upon study initiation by the study sponsor.

9.2 Packaging

Both the probiotic and the placebo capsules are packaged in identical canisters with a desiccant. Each canister contains 30 capsules.

9.3 INHP Capsule Formulation

Blends of the two strains GR-1® and RC-14® are, together with excipients, formulated into a capsule containing 180 mg of powder, 10 mg of *L. rhamnosus* GR-1® and 41 mg of *L. reuteri* RC-14® . This composition is formulated to contain a minimum of 5 billion (5.0×10^9) CFU per dose (capsule), with equal numbers of the two strains; i.e. 2.5×10^9 CFU of each of GR-1® and RC-14® at the time of manufacture. The capsules are guaranteed to contain a minimum combined total of 1×10^9 CFU throughout their shelf life.

9.4 Placebo Formulation

The composition of the gelatin capsule is 98% Gelatin and 2% Titanium dioxide. The contents of the capsule include glucose, potato starch, cellulose, and magnesium sterate.

9.5 Labelling

In order to maintain the double blind, bottles will be labelled as “J” or “K”. Each bottle will contain either GR-1® and RC-14® or placebo.

Each container will be labelled (in English and French) according to the requirements of the Natural Health Products Directorate and ICH GCP Guidelines and will include:

- Brand name or code of the Natural Health Product (NHP)/Placebo;
- A statement indicating that the NHP is an investigational NHP to be used only by a Qualified Investigator;
- The initials and subject’s identifying number;
- The expiration date;
- The recommended storage conditions;
- The lot number;
- The name and address of the manufacturer and Sponsor; and
- The protocol number.

In addition, written instructions on how to administer the INHP will be provided to the subjects.

9.6 Storage

The Investigator or designee will be responsible for ensuring that all INHP is adequately accounted for and kept in a secured area accessible only to individuals authorized to dispense the product. The capsules should be kept refrigerated at the site at 4-6ºC (±2ºC) for the duration of the study to ensure optimal stability. Once dispensed to subjects, subjects are encouraged to keep the INHP refrigerated; however, this is not a requirement. Subjects will be instructed to keep the INHP from being exposed to severe heat (e.g. a parked car on a hot day).

9.7 Accountability

All INHP provided by the Sponsor for the study and that is given to a subject must be accounted for in writing. The Investigator or designee must document receipt of all INHP at the site, inventory at the site, use by each subject, and the return of all INHP supplies to the Sponsor. These records should include dates, quantities and batch/lot/serial numbers of the INHP assigned to the subjects.

The sites must remind the subjects to return treatment containers that were dispensed for the purpose of INHP accountability. All returned containers (used and unused) may not be dispensed to another subject.

All INHP containers (used and unused) and corresponding accountability forms must be returned to the Sponsor at the end of the study.

9.8 Subject Compliance

Subject compliance that is not 100% must be properly documented. Any temporary or permanent discontinuations in INHP must be reported, specifying the date and time of missed administrations, the date of re-administration and the reason for discontinuation. Proper instructions on how to insert the INHP must be given to each subject as often as needed.

Subjects should be instructed to take the INHP at the same times twice daily, once in the morning and once in the evening at about 12 hour dose interval. If a dose is missed, that dose should be taken as soon as possible provided it is taken at least 6 hours prior to the following scheduled dose time. If more than two doses are missed, the subject will be discontinued from the study.

10 CONCOMITANT MEDICATIONS AND TREATMENTS

10.1 Rescue Medication for BV

Currently, antibiotics represent the treatment of choice for BV and these may be administered either orally or topically as detailed below:

Oral: Metronidazole 500 mg twice daily for 7 days;
Topical: Clindamycin 2% cream, 5 g daily for 7 days.

At any time during the study where a subject presents with a Nugent Score of 7 – 10, the subject will be treated for BV as deemed appropriate by the treating physician. Subjects requiring antibiotic treatment will be discontinued from the study.

10.2 Permitted Medications and Treatments

Any medication and/or treatments in use at the time of the Day 1 visit for any inter-current illness should remain constant for the duration of the study, as evaluated by the Investigator.

10.3 Medications and Products not Permitted During the Study

Any form of antibiotic is forbidden during the course of this study. The subject will be discontinued from the study if antibiotic is given to treat any medical conditions. In the event of a Nugent Score greater than or equal to seven (7), the subject will be discontinued from the study and prescribed an antibiotic as evaluated by the Investigator. Vaginal lubricants and any product applied vaginally are not permitted during the study.

11 SAFETY REPORTING

Adverse Events are defined using the ICH Clinical Safety Data Management (ICH E2A) definition.

11.1 Adverse Event

An adverse event is any untoward medical occurrence in a subject administered an INHP at any dose, which does not necessarily have a causal relationship with this treatment. An adverse event can therefore be any unfavourable and unintended sign, symptom, or disease temporally associated with the use of an INHP, whether or not related to the INHP.

For all adverse events, an assessment must be made of the seriousness, intensity and relationship to the administration of the INHP.

11.2 Serious Adverse Event

A serious adverse event (SAE) is defined as any untoward medical occurrence that at any dose:

- Results in death;
- Is life-threatening;
- Requires inpatient hospitalization or prolongation of existing hospitalization;
- Results in persistent or significant disability/incapacity; or
- Is a congenital anomaly/birth defect.

Medical and scientific judgement should be exercised in deciding whether expedited reporting is appropriate in other situations, such as important medical events that may not be immediately life-threatening or result in death or hospitalization but may jeopardize the subject or may require intervention to prevent one of the outcomes listed in the definition above. These should also usually be considered serious.

A life-threatening event is any event in which the subject was at risk of death at the time of the event; it does not refer to an event, which hypothetically might have caused death if it were more severe.

A serious unexpected adverse reaction is any serious adverse reaction that is not identified in nature, severity or frequency in the risk information set out on the label of the natural health product.

11.3 Reporting of Serious Adverse Events

All serious adverse events fulfilling the definition of seriousness and occurring during the clinical study (i.e. following informed consent and up to the end of the additional follow-up period) must be reported immediately (*within 24 hours of the Investigator's awareness*) to Integrated Research Inc. Serious Adverse Events occurring before the INHP or placebo is taken will not be captured.

In case of a serious adverse event, please complete the Serious Adverse Event (SAE) Form provided for the study and fax it immediately to:

Attn: Sherene Guindi
Integrated Research Inc.
Fax: (514) 683-0121

The Investigator must complete the SAE Form by providing at least: protocol identification, subject's initials and number, date of birth, period of the INHP use, nature of the adverse event and the relationship to the INHP.

The cause of death of a subject in a study should be reported as a serious adverse event. Death should not be reported as an adverse event, but as an outcome. The only exception is *sudden death* (when the cause of death is unknown), which is reported as a serious adverse event with death as the outcome.

In case of pregnancy during the clinical study, the subject should be discontinued from the study and the event reported. The outcome of the pregnancy should also be reported.

Unexpected adverse event is an adverse event of which the nature or severity is not consistent with the applicable product information.

In accordance with the ICH GCP Guidelines and Natural Health Products Regulations, the Sponsor will report all serious adverse NHP reactions to the Natural Health Product Directorate (NHPD) and local Ethics Committee.

12 SUBJECT WITHDRAWAL

Subjects have the right to withdraw from the study at any time and for any reason. In the event that a subject withdraws, every effort must be made to obtain "End-of-Treatment" information by completing, within the shortest delay, the assessments and procedures planned at Visit 8 – Day 47. The reason(s) for withdrawal must be described, and if more than one is given, the Investigator must specify the primary reason.

The Investigator will maintain the subject in the study as long as possible. However, the Investigator may also decide to withdraw a subject from the study in any of the following circumstances apply:

- Subject no longer meets the inclusion or exclusion criteria;
- Nugent Score less than three (3) or greater than six (6);
- Occurrence of an adverse event for which, in the opinion of the Investigator, the continuation of the study may compromise the safety of the subject; and
- Pregnancy.

In the case of withdrawal due to an adverse event which may potentially be related to the investigational products, the subject should be followed up until all abnormalities return to acceptable levels or stabilize. All adverse events must be documented on the corresponding pages in the case report form.

Any premature termination of treatment must be reported immediately to the Sponsor's designated representative.

13 STATISTICS

13.1 Statistical Methods

A complete statistical analysis plan will be finalized prior to the enrolment of the first subject into the study. All analyses and summaries, in table and graph form, will be presented by treatment group, unless specified otherwise.

Frequency distributions (n, proportions) will be used to present categorical data such as Nugent Scores and symptoms. Descriptive statistics such as n, mean, minimum, maximum, median and standard deviation (SD) will be used to present continuous variables such as age. No statistical sample size testing was performed.

13.1.1 Analysis Populations

Safety Set: All randomized subjects who received the INHP at any dose or placebo, will be included in the safety analyses.

Full Analysis Set: All randomized subjects will be analyzed following the principle of intention-to-treat (ITT) provided they received the INHP or placebo and had at least one efficacy observation after first insertion.

Per protocol Set: All subjects who are compliant with the study protocol (i.e., who do not experience any major protocol deviations), who received the INHP or placebo and had at least one efficacy observation after first insertion.

The primary efficacy analysis will be based on the Full Analysis Set, although a secondary analysis will also be performed based upon the Per-Protocol Set (if there are differences) to assess the sensitivity of the analysis to the choice of analysis population.

The analyses of all other efficacy endpoints will be based upon the Full Analysis Set only. All safety analyses will be based upon the Safety Set.

13.1.2 Efficacy Analysis

Efficacy endpoints shall include the Nugent Scores and symptoms of itching, irritation, and general vaginal discomfort, as well as other symptoms.

13.1.2.1 Nugent Score Analysis

H_o: $\pi_A = \pi_P$

H_a: $\pi_A \neq \pi_P$

π_A denotes proportion of subjects used intravaginal probiotic capsules had shown improvement in Nugent Score from intermediate (4-6) to normal status (0-3)

π_P denotes proportion of subjects used intravaginal placebo capsules had shown improvement in Nugent Score from intermediate (4-6) to normal status (0-3)

McNemar's test will be utilized to test the hypothesis at 5% level of significance, 2-sided.

13.1.3 Exploratory Analyses

Exploratory endpoints shall include the pH analyses, symptoms such as itching, irritation, and general vaginal discomfort (as well as other symptoms), microbiota analysis, microarray analysis, and multiplex immunological analysis.

13.1.3.1 Additional Nugent Score Analyses

Change in Nugent Score from baseline (no treatment phase) through the treatment phases and follow-up will be summarized and compared between the test articles.

13.1.3.2 pH Analysis

Change in pH measurement from baseline (no treatment phase) through the treatment phases and follow-up will be summarized and compared between the test articles.

13.1.3.3 Symptom Analysis

Diary questionnaire information from baseline (no treatment phase) through the treatment phases and follow-up will be summarized and compared between the test articles.

13.1.3.4 Microbiota Analysis

Abundance values will be defined as operational taxonomic units (OTUs). In order to compare microflora diversity, Shannon's diversity index[63] will be calculated for each subject at each treatment phase as the abundance values using OTUs. The Shannon's diversity index (S) is defined as $S = -\sum_{i=1}^{119} p_i \log(p_i)$ where p_i denotes the proportional abundance values at the ith OTU. The lactobacillus ratio will be calculated for each subject at each treatment phase, as the ratio of total abundance value for the lactobacillus OTUs to the total abundance value for all the OTUs.

To determine if the linear trends in the S and lactobacillus ratio trajectories differ significantly across subjects and treatment phases, the following linear mixed effect model[64] will be applied to the data $y_{it} = \mu + a_i + b_i t + \epsilon_{it}$. The fixed effect being the intercept only and random effects being subject intercepts and slopes where y_{it} denoted the S value of subject i at time point t and μ is an unknown fixed constant, a_i is a random subject intercept, b_i a random subject slope, and a_i, b_i, ϵ_{it} are independent normal random variables.

In order to determine if a statistical relationship existed between microbial diversity and vaginal symptoms, a weighted least squares analysis[65] will be applied to the data. Severity of vaginal symptoms will be treated as ordinal values 0, 1, 2, and 3, for example. The weighted least

squares model $\overline{y}_{id} = \mu_0 + \mu_1 d + \epsilon_{id}$ will be used to test the linear effect of vaginal symptoms with respect to microbial diversity. Where $\overline{y}_{id}$ denotes the average *S* of subject *i* at dryness *d*. The quantities μ_0 and $\mu_1 d$ are constants representing intercept and slope respectively.

13.1.3.5 Microarray Analysis

mRNA will be extracted from vaginal epithelial cells and gene expression changes determined using a human GeneChip array (Affymetrix, Santa Clara, CA). Probe level data will be generated using Affymetrix Command Console v1.1. Probes will be summarized to gene level data in Partek Genomics Suite v6.5 (Partek, St. Louis, MO) using the Robust Multi-array Analysis (RMA) algorithm (Irizarry, 2003). A list of genes will be filtered to only include genes differentially expressed by at least 2-fold between treatment phases within an individual. Analysis of functional networks will be carried out with Ingenuity Pathway Analysis 8.5 Software (Ingenuity Systems, Redwood city, CA, USA).

13.1.3.6 Metabolomic Analysis

The metabolomic samples will be analyzed by GC-MS, LC-MS and NMR to determine small molecule profiles in the vaginal cavity. Molecules such as short chain fatty acids, metabolic intermediates, hormones and other signaling molecules can be detected using these techniques. The small molecule profile will be compared between treatments within a subject and can be used to provide information on the biological changes associated with probiotic treatment.

13.1.3.7 Multiplex Immunological Analysis

Levels of immunological cytokines will be measured using a multiplex antibody based approach. Detection of specific cytokines will be analyzed using flow cytometry based techniques. The cytokines will be quantitated and levels compared between treatments.

13.1.4 Safety Analysis

Safety analysis will be completed utilizing the data in the Safety Set as defined in Section 13.1.1.Adverse events occurring during the study and follow-up will be tabulated by Body System, severity, and Investigator reported relationship to the INHP. Any deaths or serious INHP associated AE cases will be summarized.

13.2 Sample Size and Power Considerations

No formal sample size calculation was performed for this study. The number of subjects to be randomized is based on feasibility and practical considerations for such an exploratory study. It is planned that a total of fourteen (14) subjects will be randomized in this study.

14 STUDY MANAGEMENT

14.1 Protocol Amendments

The Investigator should not implement any deviation from, or changes to, the protocol without agreement by the Sponsor and prior review and documented approval or favourable opinion of an amendment from the Ethics Committee and NHPD, except where necessary to eliminate an immediate hazard to study subjects, or when the change involves only logistical or administrative aspects of the study (change of monitor, change of telephone number, etc.).

14.2 Subject Confidentiality

The Investigator and the Sponsor are responsible for ensuring that subjects' confidentiality will be maintained. Case report forms or other study documents will identify subjects by initials, identification number, and not by name. The Investigator will keep a separate confidential subject identification log showing initials, identification numbers, names and addresses. All documents showing the subject's identity will be kept in strict confidence by the Investigator. Authorized individuals participating in the monitoring or audit visit at sites are submitted to the responsibility for subjects' confidentiality.

14.3 Quality Control and Quality Assurance Procedures

The Sponsor or Sponsor's representative will monitor the study at regular intervals. At each monitoring visit, the case report forms will be verified against the source documents to ensure adherence to the protocol and compliance of the site to the ICH GCP Guidelines. The monitor will also review the INHP and rescue medication records, regulatory, essential and study documents and correspondence. The monitor will periodically inspect the facilities to ensure that the site's personnel and facilities remain adequate for the duration of the study.

The sites may be audited at any time during the study or thereafter by the quality assurance personnel of the Sponsor, or inspected by the regulatory authorities.

14.4 Criteria for Early Termination of the Study

The Sponsor reserves the right to terminate the study at any time, e.g. if the variables used to assess the safety data of the INHP clearly show that it would be unethical to continue the study.

14.5 Discontinuation of the Study

The Sponsor may decide to discontinue the study prematurely at a specific site for the following reasons:

- Significant changes in site personnel, technical environment or management, which may affect further potential and compliance with ICH GCP Guidelines;
- Non-compliance with protocol, which results in major violation;
- Evidence of fraud;
- Significant change of benefit/risk ratio for the subjects, based on related adverse event occurrence; and
- Significant changes in justification/usefulness of the study.

15 ETHICS

15.1 Good Clinical Practice

This study is to be conducted in accordance with the protocol and principles of Good Clinical Practice as stated in the ICH GCP Guidelines, Declaration of Helsinki and local regulations.

The Investigator is responsible for ensuring to conduct the study in compliance with the protocol and principles of Good Clinical Practice as stated above.

15.2 Ethics Committee

The protocol including the complete appendices and any amendments will be submitted to the appropriate Ethics Committee for review and approval before the start of the study. Any amendment made to the approved protocol must be forwarded to and approved by the same Ethics Committee before its implementation and transmitted to the regulatory authorities, as required by local regulations.

The Ethics Committee must be informed of all significant changes to the protocol for review and approval and of all serious or unexpected adverse events occurring during the study, which are likely to affect the safety of the subjects or the conduct of the study.

15.3 Informed Consent

The Investigator or designee should fully inform the subject of all pertinent aspects of the study. The study will be explained verbally as well as on the Information Sheet and Informed Consent Form and the Consent for Donation and Storage of Patient Samples for Research Tests form. The subjects must be given ample opportunity to inquire about details of the study. The subjects must be given adequate opportunity to read and understand the Information Sheet and Informed Consent Form before being involved in the study. Each subject must sign and date the Information Sheet and Informed Consent Form, and must receive a copy of the document. Each subject's original signed Informed Consent Form must be kept on file by the Investigator and will be verified by the monitor and possibly inspected by regulatory authorities, the Sponsor or its designee. The Site Specific Information Sheet and Informed Consent Form will be submitted for Ethics Committee approval as a separate document.

Written informed consent must be obtained before any study-related actions begin. The Information Sheet and Informed Consent Form and any other written information provided to the subjects should be revised whenever new information that may be relevant to the subject's consent becomes available. The revisions of these documents should be reviewed and approved by the Ethics Committee prior to implementation.

15.4 Direct Access to Source Data/Documents

By signing the protocol the Investigator agrees to conduct the study in accordance with the local legal requirements and to fully cooperate by allowing access to all documents by authorized individuals. This may include an audit by the Sponsor or designee, request by the Ethics Committee and inspection by regulatory authorities at any time. Direct access to source documents by the Sponsor, designated individuals or regulatory inspectors must be granted by the Investigator.

15.5 Data Handling and Record Keeping

The Investigator is responsible for ensuring that all data are recorded in the case report form provided by the Sponsor. Data should be entered promptly, using black ballpoint pens, and should be accurate, complete and legible.

Data reported in the case report form should be consistent with the source documentation; all discrepancies should be explained. "Source documentation" consists of all original documents, data and records (e.g., hospital charts, clinic or office charts, radiographs, laboratory reports, pharmacy records, subject diaries).

All original reports must be available for verification whenever necessary by the Sponsor or its representatives, regulatory authorities or Ethics Committee without affecting the confidentiality of the subjects.

Any change or correction to a case report form should be dated, initialled and explained (if necessary) and should not obscure the original entry. The words or figures should be crossed through with a single line. The Investigator must sign the signature page of each visit of the case report form for each subject to indicate that he/she has verified all the data for completeness and accuracy.

Original completed case report forms will be collected by the monitor and copies will be retained at the site along with the supporting source documents. Copies of laboratory reports and other relevant documents may be collected by the monitor. In these cases, the subject's identification will be kept confidential and will be limited to initials and identification number.

All study-related records, including source documents, case report forms and regulatory, essential and study documents must be retained for at least 25 years as required by the Canadian regulatory authorities. These documents should be retained for a longer period if required by regulatory agency or by an agreement with the Sponsor. The Investigator is responsible for the retention of all study documents for the appropriate period, and must inform the Sponsor in writing of any change in the status of these documents. The Sponsor will inform the Investigators when these documents will no longer need to be retained.

16 PUBLICATION POLICY

The Investigators agree to keep all information provided by the Sponsor and all data collected during the study confidential. No presentation or publication of the study results may be made without prior approval by the Sponsor.

17 REFERENCES

1. Nyirjesy P. Vulvovaginal candidiasis and bacterial vaginosis. Infectious Disease Clinics of North America. 2008 2008 Dec;22(4):637-52.
2. Lipsky MS, Waters T, Sharp LK. Impact of vaginal antifungal products on utilization of health care services: evidence from physician visits. Journal of the American Board of Family Practice. 2000 May-Jun;13(3):178-82.
3. Anderson MR, Klink K, Cohrssen A. Evaluation of vaginal complaints. JAMA. 2004 Mar 17;291(11):1368-79.
4. Cherpes TL, Meyn LA, Krohn MA, Lurie JG, Hillier SL. Association between acquisition of herpes simplex virus type 2 in women and bacterial vaginosis. Clinical Infectious Diseases. 2003 Aug 1;37(3):319-25.
5. Goldenberg RL, Culhane JF, Iams JD, Romero R. Epidemiology and causes of preterm birth. The Lancet. 2008;371(9606):75-84.
6. Cohen MS. Sexually transmitted diseases enhance HIV transmission: no longer a hypothesis. Lancet. 1998;351 Suppl 3:5-7.
7. Klebanoff MA, Schwebke JR, Zhang J, Nansel TR, Yu K-F, Andrews WW. Vulvovaginal symptoms in women with bacterial vaginosis. Obstetrics & Gynecology. 2004 Aug;104(2):267-72.

8. Sobel JD, Ferris D, Schwebke J, Nyirjesy P, Wiesenfeld HC, Peipert J, et al. Suppressive antibacterial therapy with 0.75% metronidazole vaginal gel to prevent recurrent bacterial vaginosis. American Journal of Obstetrics & Gynecology. 2006 May;194(5):1283-9.
9. Allsworth J.E, Peipert J.F. Prevalence of bacterial vaginosis 2001–2004 national health and nutrition examination survey data. Obstet Gynecol. 2007;109:114–20.
10. Cauci S, Driussi S, De Santo D, Penacchioni P, Iannicelli T, Lanzafame P, et al. Prevalence of bacterial vaginosis and vaginal flora changes in peri- and postmenopausal women. Journal of Clinical Microbiology. 2002 Jun;40(6):2147-52.
11. Cribby S, Taylor M, Reid G. Vaginal microbiota and the use of probiotics. Interdisciplinary Perspectives on Infectious Diseases. 2008:1-9.
12. Nyirjesy P. Postmenopausal vaginitis. Current Infectious Disease Reports. 2007;9:480–4.
13. Chan RC, Bruce AW, Reid G. Adherence of cervical, vaginal and distal urethral normal microbial flora to human uroepithelial cells and the inhibition of adherence of gram-negative uropathogens by competitive exclusion. Journal of Urology. 1984 Mar;131(3):596-601.
14. Petricevic L, Unger FM, Viernstein H, Kiss H. Randomized, double-blind, placebo-controlled study of oral lactobacilli to improve the vaginal flora of postmenopausal women. European Journal of Obstetrics, Gynecology, & Reproductive Biology. 2008 Nov;141(1):54-7.
15. Verhelst R, Verstraelen H, Claeys G, Verschraegen G, Delanghe J, Van Simaey L, et al. Cloning of 16S rRNA genes amplified from normal and disturbed vaginal microflora suggests a strong association between Atopobium vaginae, Gardnerella vaginalis and bacterial vaginosis. BMC Microbiology. 2004 Apr 21;4:16.
16. Verstraelen H, Verhelst R, Claeys G, Temmerman M, Vaneechoutte M. Culture-independent analysis of vaginal microflora: the unrecognized association of Atopobium vaginae with bacterial vaginosis. American Journal of Obstetrics & Gynecology. 2004 Oct;191(4):1130-2.
17. Keane EA, Thomas J, Gilroy B, Renton A, Taylor-Robinson D. The association of Mycoplasma hominis, Ureaplasma urealyticum and Mycoplasma genitalium with bacterial vaginosis: observations on heterosexual women and their male partners. Int J STD AIDS. 2000 June 1, 2000;11(6):356-60.
18. Hallen A, Pahlson C, Forsum U. Bacterial vaginosis in women attending STD clinic: diagnostic criteria and prevalence of Mobiluncus spp. Genitourinary Medicine. 1987 Dec;63(6):386-9.
19. Nugent RP, Krohn MA, Hillier SL. Reliability of diagnosing bacterial vaginosis is improved by a standardized method of gram stain interpretation. Journal of Clinical Microbiology. 1991 Feb;29(2):297-301.
20. Laughton JM, Devillard E, Heinrichs DE, Reid G, McCormick JK. Inhibition of expression of a staphylococcal superantigen-like protein by a soluble factor from Lactobacillus reuteri. Microbiology. 2006 Apr;152(Pt 4):1155-67.
21. Reid G. Probiotic agents to protect the urogenital tract against infection. American Journal of Clinical Nutrition. 2001 Feb;73(2 Suppl):437S-43S.
22. Osset J, Bartolome RM, Garcia E, Andreu A. Assessment of the capacity of Lactobacillus to inhibit the growth of uropathogens and block their adhesion to vaginal epithelial cells. Journal of Infectious Diseases. 2001 Feb 1;183(3):485-91.
23. Heinemann C, van Hylckama Vlieg JE, Janssen DB, Busscher HJ, van der Mei HC, Reid G. Purification and characterization of a surface-binding protein from Lactobacillus

fermentum RC-14 that inhibits adhesion of Enterococcus faecalis 1131. FEMS Microbiology Letters. 2000 Sep 1;190(1):177-80.

24. Hutt P, Shchepetova J, Loivukene K, Kullisaar T, Mikelsaar M. Antagonistic activity of probiotic lactobacilli and bifidobacteria against entero- and uropathogens. Journal of Applied Microbiology. 2006 Jun;100(6):1324-32.
25. Kim SO, Sheikh HI, Ha S-D, Martins A, Reid G. G-CSF-mediated inhibition of JNK is a key mechanism for Lactobacillus rhamnosus-induced suppression of TNF production in macrophages. Cellular Microbiology. 2006 Dec;8(12):1958-71.
26. Chiaffarinoa F, Parazzinia F, De Besic P, Lavezzari M. Risk factors for bacterial vaginosis. European Journal of Obstetrics & Gynecology and Reproductive Biology 2004;117:222–6.
27. Marrazzo JM, Koutsky LA, Eschenbach DA, Agnew K, Stine K, Hillier SL. Characterization of vaginal flora and bacterial vaginosis in women who have sex with women. Journal of Infectious Diseases. 2002 May 1;185(9):1307-13.
28. Beigi RH, Austin MN, Meyn LA, Krohn MA, Hillier SL. Antimicrobial resistance associated with the treatment of bacterial vaginosis. American Journal of Obstetrics & Gynecology. 2004 Oct;191(4):1124-9.
29. Torok MR, Miller WC, Hobbs MM, Macdonald PDM, Leone PA, Schwebke JR, et al. The association between Trichomonas vaginalis infection and level of vaginal lactobacilli, in nonpregnant women. Journal of Infectious Diseases. 2007 Oct 1;196(7):1102-7.
30. Schwebke JR, Desmond R. A randomized trial of metronidazole in asymptomatic bacterial vaginosis to prevent the acquisition of sexually transmitted diseases. American Journal of Obstetrics & Gynecology. 2007 Jun;196(6):517.e1-6.
31. Schwebke JR, Weiss HL. Interrelationships of bacterial vaginosis and cervical inflammation. Sexually Transmitted Diseases. 2002 Jan;29(1):59-64.
32. Atashili J, Poole C, Ndumbe PM, Adimora AA, Smith JS. Bacterial vaginosis and HIV acquisition: a meta-analysis of published studies. AIDS. 2008 Jul 31;22(12):1493-501.
33. Wilson J. Managing recurrent bacterial vaginosis. Sex Transm Infect. 2004;80:8-11.
34. Bradshaw CS, Morton AN, Hocking J, Garland SM, Morris MB, Moss LM, et al. High recurrence rates of bacterial vaginosis over the course of 12 months after oral metronidazole therapy and factors associated with recurrence. Journal of Infectious Diseases. 2006 Jun 1;193(11):1478-86.
35. Bradshaw CS, Tabrizi SN, Fairley CK, Morton AN, Rudland E, Garland SM. The association of Atopobium vaginae and Gardnerella vaginalis with bacterial vaginosis and recurrence after oral metronidazole therapy. Journal of Infectious Diseases. 2006 Sep 15;194(6):828-36.
36. Schwebke JR, Desmond R. Natural history of asymptomatic bacterial vaginosis in a high-risk group of women. Sexually Transmitted Diseases, November 2007, Vol 34, No 11, p876–877. 2007;34:876-7.
37. Amsel R, Totten PA, Spiegel CA, Chen KC, Eschenbach D, Holmes KK. Nonspecific vaginitis. Diagnostic criteria and microbial and epidemiologic associations. American Journal of Medicine. 1983 Jan;74(1):14-22.
38. MacPhee RA, Hummelen R, Bisanz JE, Miller WL, Reid G. Probiotic strategies for the treatment and prevention of bacterial vaginosis. Expert Opin Pharmacother. 2010;11:2985-95.
39. Pfizer-Canada. DALACIN* VAGINAL CREAM; Clindamycin Phosphate Vaginal Cream (20 mg clindamycin/g)-Product Monograph; revised 2009.

40. Sanofi-Aventis. (Metronidazole) 10% Cream, 500 mg Capsules-Product monograph; revised 2009.
41. Mission-Pharmacal-Company. Tindamax® (tinidazole) tablets for oral use-Prescribing information; revised 2007.
42. Nyirjesy P, McIntosh MJ, Steinmetz JI, Schumacher RJ, Joffrion JL. The effects of intravaginal clindamycin and metronidazole therapy on vaginal mobiluncus morphotypes in patients with bacterial vaginosis. Sexually Transmitted Diseases. 2007 Apr;34(4):197-202.
43. Fooks LJ, Gibson GR. Probiotics as modulators of the gut flora. British Journal of Nutrition. 2002;88:S39–S49
44. Burton JP, Cadieux PA, Reid G. Improved understanding of the bacterial vaginal microbiota of women before and after probiotic instillation. Appl Environ Microbiol. 2003;69:97-101.
45. Velraeds MM, van de Belt-Gritter B, van der Mei HC, Reid G, Busscher HJ. Interference in initial adhesion of uropathogenic bacteria and yeasts to silicone rubber by a Lactobacillus acidophilus biosurfactant. J Med Microbiol. 1998;47:1081–5.
46. Ekmekci H, Aslim B, Ozturk S. Characterization of vaginal lactobacilli coaggregation ability with Escherichia coli. Microbiol Immunol 2009;53:59-65.
47. Voltan S, Martines D, Elli M, Brun P, Longo S, Porzionato A, et al. Lactobacillus crispatus m247-derived H_2O_2 acts as a signal transducing molecule activating peroxisome proliferator activated receptor-g in the intestinal mucosa. Gastroenterology. 2008;135:1216-27.
48. Martins AJ, Colquhoun P, Reid G, Kim SO. Reduced expression of basal and probiotic-inducible G-CSF in intestinal mononuclear cells is associated with inflammatory bowel disease. Inflamm Bowel Dis. 2009;15:515-25.
49. Saunders S, Bocking A, Challis J, Reid G. Effect of Lactobacillus challenge on Gardnerella vaginalis biofilms. Colloids Surf B Biointerfaces. 2007;55:138-42.
50. Mastromarino P, Macchia S, Meggiorini L, Trinchieri V, Mosca L, Perluigi M, et al. Effectiveness of Lactobacillus-containing vaginal tablets in the treatment of symptomatic bacterial vaginosis. Clinical Microbiology & Infection. 2009 Jan;15(1):67-74.
51. Hallen A, Jarstrand C, Pahlson C. Treatment of bacterial vaginosis with lactobacilli. Sexually Transmitted Diseases. 1992 May-Jun;19(3):146-8.
52. Rossi A, Rossi T, Bertini M, Caccia G. The use of Lactobacillus rhamnosus in the therapy of bacterial vaginosis. Evaluation of clinical efficacy in a population of 40 women treated for 24 months. Arch Gynecol Obstet. 2010;281:1065-9.
53. Cannon JP, Lee TA, Bolanos JT, Danziger LH. Pathogenic relevance of Lactobacillus: a retrospective review of over 200 cases. European Journal of Clinical Microbiology & Infectious Diseases. 2005 Jan;24(1):31-40.
54. Chr. Hansen A/S. Investigator's Brochure. Capsule containing Lactobacillus rhamnosus GR-1® and Lactobacillus reuteri RC-14®. Edition 01; release date 2011 (Apr)
55. Ahrne S, Noback S, Jeppsson B, Adlerberth I, Wold AE, Molin G. The normal Lactobacillus flora of healthy human rectal and oral mucosa. J Appl Microbiol. 1998;85:88-94.
56. Mogensen G, Salminen S, O'Brien J, Ouwehand A, Holzapfel W, Shortt C, et al. Inventory of microorganisms with a documented history of use in food. Bull. Int. Dairy Fed. 2002:10-19.

57. Anukam KC, Duru MU, Eze CC, Egharevba J, Aiyebelehin A, Bruce A, et al. Oral use of probiotics as an adjunctive therapy to fluconazole in the treatment of yeast vaginitis: A study of Nigerian women in an outdoor clinic. Microb Ecol Health Dis. 2009;21:72-77.

58. Anukam K, Osazuwa E, Ahonkhai I, Ngwu M, Osemene G, Bruce AW, et al. Augmentation of antimicrobial metronidazole therapy of bacterial vaginosis with oral probiotic Lactobacillus rhamnosus GR-1 and Lactobacillus reuteri RC-14: randomized, double-blind, placebo controlled trial. Microbes Infect. 2006;8:1450-1454.

59. Martinez RC, Franceschini SA, Patta MC, Quintana SM, Gomes BC, De Martinis EC, et al. Improvied cure of bacterial vaginosis with single dose of tinidazole (2 g), Lactobacillus rhamnosus GR-1, and Lactobacillus reuteri RC-14: a randomized, double-blind, placebo-controlled trial. Can J Microbiol. 2009b;55:133-138.

60. Hummelen R, Changalucha J, Butamanya NL, Cook A, Habbema JDF, Reid G. Lactobacillus rhamnosus GR-1 and L. reuteri RC-14 to prevent or cure bacterial vaginosis among women with HIV. International Journal of Gynecology & Obstetrics. 2010;111(3):245-8.

61. Danielsen M, Wind A. Susceptibility of Lactobacillus spp. to antimicrobial agents Int J Food Microbiol. 2003;82:1-11.

62. Larsen CN, Nielsen S, Kaestel P, Brockmann E, Bennedsen M, Christensen HR, et al. Dose-response study of probiotic bacteria Bifidobacterium animalis subsp lactis BB-12 and Lactobacillus paracasei subsp paracasei CRL-341 in healthy young adults. Eur J Clin Nutr. 2006;60:1284-1293.

63. Magurran, AE. Ecological Diversity and its Measurement. Princeton University Press, Princeton, U.S.A. 1988:100-130.

64. Laird, NM, Ware JH. Random-Effects Models for Longitudinal Data. Biometrics 1982; 38:963-974.

65. Agresti, L. Categorical data analysis. John Wiley & Sons, Inc., Hoboken, New Jersey. 2002:600-619.
